# Parameterization of cell-free systems with time-series data using KETCHUP

**DOI:** 10.1371/journal.pcbi.1013724

**Published:** 2025-11-21

**Authors:** Mengqi Hu, Syed Bilal Jilani, Daniel G. Olson, Costas D. Maranas

**Affiliations:** 1 Department of Chemical Engineering, The Pennsylvania State University, University Park, Pennsylvania, United States of America; 2 Thayer School of Engineering, Dartmouth College, Hanover, New Hampshire, United States of America; University of Southern California, UNITED STATES OF AMERICA

## Abstract

Kinetic models mechanistically link enzyme levels, metabolite concentrations, and allosteric regulation to metabolic reaction fluxes. This coupling allows for the quantitative elucidation of the dynamics of the evolution of metabolite concentrations and metabolic fluxes as a function of time. So far, most large-scale kinetic model parameterizations are carried out using mostly steady-state flux measurements supplemented with metabolomics and/or proteomics data when available. Even though the parameterized kinetic model can trace a temporal evolution of the system, lack of anchoring to temporal data reduces confidence in the dynamics predictions. Notably, the simulation of enzymatic cascade reactions requires a full description of the dynamics of the system as a steady-state is not applicable given that all measured metabolite concentrations vary with time. Here we describe how kinetic parameters fitted to the dynamics of single-enzyme assays remain accurate for the simulation of multi-enzyme cell-free systems. Herein, we demonstrate two extensions for the Kinetic Estimation Tool Capturing Heterogeneous datasets Using Pyomo (KETCHUP) software tool for parameterizing a kinetic model of the cell-free kinetics of formate dehydrogenase (FDH) and 2,3-butanediol dehydrogenase (BDH) through the use of time-course data across various initial conditions. An implemented extension of KETCHUP allowing for the reconciliation of measurement time-lag errors present in datasets was used to parameterize kinetic models using multiple datasets. By combining the kinetic parameters identified by the FDH and BDH assays, accurate simulation of the binary FDH-BDH system was achieved.

## Author summary

Metabolic engineering of microorganisms offers a sustainable and renewable alternative for industrial scale production of commodity chemicals. Large-scale metabolic stoichiometric models enable means to elucidate an organism’s physiological behavior towards both environment and genetic perturbations and predict optimal interventions to maximize production. Kinetic models extend stoichiometric models’ capabilities in characterizing metabolic phenotypes by mathematically linking enzyme catalyzed reactions in metabolic networks as functions of metabolite concentrations, enzyme levels, and allosteric regulations. Although these kinetic descriptions improve predictive accuracy and strain design, the lack of enzyme information, raw experimental data, and high computational cost preclude the construction and parameterization of detailed large-scale kinetic models. Here, we introduce an extension of KETCHUP, an open-source semi-automatic kinetic construction and parameterization tool that utilizes steady-state data as a pilot for the parameterization of single-enzyme cell-free time-course data. We show that this tool offers comparable performance to existing tools during parameterization of single enzymes and successful recapitulation of metabolite profiles for a two-enzyme system when simulating a kinetic model with parameters from the two single-enzymes. This study showcases a high throughput pipeline that can be integrated into our framework for larger scaled networks.

## Introduction

Advances in metabolic engineering has led to many successful proof-of-concept designs and experiments for the renewable production of numerous chemicals and commodities such as biofuels [1–3], pharmaceuticals [4,5], isoprenoids [6–8], fatty acids [9,10], and organic acids [11,12]. However, the commercialization of these strain designs is hindered by low titer and yields which requires pathway optimization for scalable production [13]. These issues arise from factors such as *(i)* inherent complexities of metabolic and regulatory networks which hinder optimal and simple strain designs, *(ii)* non-native biosynthetic pathways that affect cell viability and homeostasis [14,15], and *(iii)* lack of detailed kinetic information for an organism’s key enzymes. While a “whole cell” strain is often the end goal for most metabolic engineering projects, cell-free systems (CFS) can contribute to overcoming bottlenecks during the strain and pathway design process. CFSs are fundamentally different than “whole cells” as the homeostasis requirement of living cells and most forms of protein-level feedback can be circumvented allowing for proper diagnosis of kinetic information [15]. The lack of compartmentalization results in a dilute and well-mixed reaction environment allowing for high resolution in the observation of reaction kinetics [16]. This ease of experimental design can facilitate the discovery of regulatory interactions and elucidation of resource allocations [17]. The lack of enzyme self-regeneration though requires estimation of enzyme stability to reliably elucidate catalytic rates [18].

CFS can be divided into two types based on the method of preparation: (*i*) cell-free protein synthesis (CFPS) systems (crude extract-based and reconstituted), and (*ii*) purified enzyme-based systems [19]. The extract-based CFPS was first used for bio-ethanol production from fermenting sugar using cell extracts from yeast [20] (crude extract-based systems) and later used to study the dynamics in protein synthesis where researchers engineer (*i.e.,* DNA sequences for expression) and assembled components required for protein synthesis reactions (*i.e.,* amino acids, energy buffers and cofactors) into a cellular lysate (*i.e.,* cytoplasmic lysate from living cell that only contains transcription and translation machinery for protein synthesis). Later, the first reconstituted system named Protein synthesis Using Recombinant Elements (PURE) system [21] was developed, where only purified factors essential for protein synthesis (*e.g.,* tRNAs, ribosome, proteins) are used. This system provided advantages over its crude extracts counterpart such as (*i*) precise composition, (*ii*) a stable and deterministic system, and (*iii*) flexible gene expression or expansion [22]. These advantages have facilitated studies on protein expression and folding [23,24] and improved protein production resulting from lack of protease and nucleases [25]. However, reconstituted systems face challenges in scalability over crude extracts resulting from higher costs (*i.e.,* costs between PURE and crude extract reactions) and lower yields [26]. Nevertheless, CFPS is arguably the most widely used CFS technology with successful scale-up pilot studies [27,28] and high-throughput proteomics [29,30]. This cell-free metabolic engineering platform has demonstrated comparable performance with cell-based systems [31], especially in the synthesis of biologic therapeutics (*e.g.*, antibodies [32], antimicrobial peptides [33], or vaccines [34]). The key advantage of CFPS cell-free systems over cell-based systems is that they are unconstrained by homeostatic considerations, allowing for continuous probing over a specified time horizon. Alternatively, enzyme-based CFSs use purified enzymes for single reaction or pathway (*i.e.,* cascade reaction) exploratory purposes. This set-up allows for flexible engineering and complete control of the reaction parameters (*i.e.,* user-defined enzyme and metabolite concentrations) over CFPS systems which has competing reactions and toxic cofactors that hinder the targeted metabolic reaction [35,36]. For example, a system of 27 purified enzymes has been shown to convert glucose to terpenes [37] or a system of three enzymes was used to produce UDP-N-acetylglucosamine from its direct precursor N-acetylglucosamine [38]. These applications demonstrate that both CFPS and enzyme-based CFSs can be valuable tools for the exploration of alternative pathway designs. However, the number of experimental variables (*i.e.,* initial concentrations of enzymes, substrates, and cofactors) can make it difficult to optimize performance empirically, and in some cases, small changes in initial conditions can prematurely halt the reaction. For example, enzyme cascades that use the glycolysis pathway are highly sensitive to the initial hexokinase concentration [37]. Thus, there is a need to develop predictive computational models to assist in optimizing system performance and to test hypothesis for system malfunctions.

Multiple computational methods and models have been used for the elucidation of metabolism. The earliest methods are constraint-based methods such as flux balance analysis [39] where an organism’s phenotype is simulated based on cellular objectives (*i.e.,* growth). Unfortunately, stoichiometric models cannot explicitly relate enzyme information or regulatory processes (*e.g.*, inhibition), limiting the advantages afforded by CFSs (*i.e.,* qualitative dynamic data that provides vital insight towards enzyme metabolism). Kinetic metabolic models address these shortcomings by incorporating enzyme kinetics, allosteric regulatory interactions, and enzyme and metabolite levels into reaction fluxes by offering a more comprehensive description of cell metabolism than stoichiometric models alone. These mathematical models offer a more comprehensive description of cell metabolism [40,41] than stoichiometric models and improves predictive capabilities [42–45], strain designs [46–48], and elucidation of regulatory interactions [49–51]. Several frameworks have been developed to facilitate the parametrization of large-scale kinetic models from a top-down (*e.g.*, elementary reaction steps [52], Log-Lin formalism [53]) approach by using generalized rate laws for reactions due to lacking enzyme mechanism information. However, this generalized approach obscure details on individual enzyme mechanisms (*e.g.,* substrate/product binding/release order), especially for allosteric regulations. For example, glucose 6-phosphate dehydrogenase binds substrates sequentially [54] whereas transketolases follow a ping-pong mechanism [55]. Despite the lack of specific enzyme information, these approaches of parameterizing kinetic models with *in vivo* datasets have successfully provided sufficiently quantitative description of cellular processes [56].

Contrary to large-scale kinetic models that commonly use a “top-down” approach for parameterization by modeling “whole cells” with steady-state *in vivo* kinetic data, small-scale kinetic models that capture transient metabolism in higher resolution require a “bottom-up” approach. These small-scale models typically are used to study cell-free systems containing either a singular enzyme or cascading enzymes that constitute the pathway of interest [57,58]. Cell-free systems provide a straightforward environment, devoid of interactions from the rest of the cellular components, for the characterization of specific enzyme activities and mechanisms. This trait allows for the construction and validation of various mechanistic models [59] and reconcile information between in vitro and in vivo enzyme functionality [60]. Moreover, combining advances in rapid prototyping of individual enzymes [61,62] in CFSs and efficient parameterization algorithms help speed-up the construction of high quality large-scale kinetic models.

The construction and parameterization of detailed large-scale kinetic models are often met with challenges such as: *(i)* computational cost in solving nonlinear mechanistic rate laws, *(ii)* algorithmic challenges in fitting of dataset(s) to a global or suitably low local minimum, *(iii)* paucity of available *in vivo* fitting data, and *(iv)* lack of knowledge for specific enzyme mechanisms. For large-scale kinetic model parameterization, several frameworks [51–53,63–65] have been proposed to address the first three challenges to various extent. Alternatively, for elucidation of enzyme kinetics, tools have been developed for custom rate laws selections but lack grouping of fitting datasets for parameterization [66–69] (*e.g.,* modifications on enzyme levels corresponding to fitting datasets). To reduce computational burden and better utilize available *in vivo* datasets, Optimization and Risk Analysis of Complex Living Entities (ORACLE) [53] and Metabolic Control Analysis (MCA) [70] utilizes Log-lin kinetic rate laws to linearize the system of nonlinear equations through first-order approximations whereas Mass Action Stoichiometric Simulation (MASS) [65], Ensemble Modeling (EM) [52], General Reaction Assembly and Sampling Platform (GRASP) [71], and Kinetic-based Fluxomics Integration Tool (K-FIT) [63] decomposes metabolic reactions into elementary steps that follow mass action kinetics. These five frameworks typically parameterize kinetic models with *in vivo* datasets (*i.e.,* combinations of fluxomics, proteomics, and/or metabolomics) to avoid potential discrepancies between *in vivo* and *in vitro* kinetic parameters, especially for turnover numbers (*i.e.*, both apparent turnover, k_app_ and standard turnover, k_cat_ values for both prokaryotes [72] and eukaryotes [73]). Fortunately, studies on CFSs that mimic *in vivo* environments [74,75] offer the means to bridge the gap between *in viv*o and *in vitro* kinetic parameter values [76] and provide detailed enzyme mechanism and kinetic information, addressing the fourth challenge of detailed large-scale kinetic model construction [77]. However, most kinetic model development tools automatically *(i)* use only a single uniform rate law formalism for all reactions, *(ii)* accept limited select datasets, *(iii)* and/or is unscalable resulting in long parameterization times and/or low convergence of a stable solution. For example, K-FIT automatically assign all metabolic reactions into elementary reactions using mass action kinetic rate laws while only accepting fluxomics datasets of reference and perturbation states. On the other hand, COPASI [67] allows the user to set the rate law from a restrictive adaptive preset selection number of participating metabolites increase for the reaction. Tellurium [68] and SKiMpy [69] allow for customized kinetic rate laws but require direct integration of systems of ordinary differential equations, which hamper model network scalability [63,78].

Scalability and rate law flexibility motivated the development of the open-sourced Kinetic Estimation Tool Capturing Heterogeneous datasets Using Pyomo (KETCHUP) which semi-automatically constructs and parameterizes kinetic models [79] using the interior point optimizer IPOPT. The original implementation of KETCHUP was benchmarked against previous kinetic models parameterized with steady-state data for groups of genetically perturbed strains; it demonstrated improved parameterization times and quality of fit compared to their respective previous parameterization tool. Moreover, KETCHUP highlights the capability of parameterizing kinetic models with multiple types of flux and metabolite concentration datasets simultaneously. In this work, we extend KETCHUP’s parameterization pipeline to the use of time-course data, enabling future KETCHUP large-scale kinetic model parameterization to simultaneously fit both dynamic and steady-state datasets. This update streamlines user-defined custom rate laws as inputs and exposes this to the graphical user interface. To demonstrate this new extension, we experimentally collected and parameterized two separate kinetic models using *in vitro* time-course data from CFS for formate dehydrogenase (FDH) from *Candida boidinii* and 2,3-butanediol dehydrogenase (BDH) from *Serretia marcescens.* A KETCHUP extension is used to identify systematic experimental errors arising from time-delayed measurements and propose lag-times needed to be imposed on the data to enable correct recapitulation of the fitted data. By integrating separately parameterized FDH and BDH kinetic parameter values, the experimental measured metabolite profiles for the two enzyme cell-free system FDH-BDH are recapitulated. These two extensions allow for systematic processing and filtering of raw data and streamline the incorporation of dynamic data into KETCHUP’s workflow (which previously only automatically accepts steady-state data), enabling parameterizations of large-scale kinetic models using steady-state and dynamic data simultaneously along with heterogenous datasets (*i.e.,* fluxomics, proteomics, and metabolomics).

## Results

### Overview

**Fig 1:**
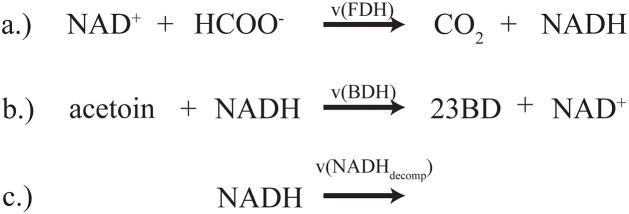
Reaction mechanisms for a) formate dehydrogenase (FDH), b) 2,3-butanediol dehydrogenase (BDH), and c) NADH decomposition reactions. Protons are excluded in the kinetic model and reflected in this schematic for BDH. Rates (v) for each reaction are defined in section “model construction”.

**Fig 2:**
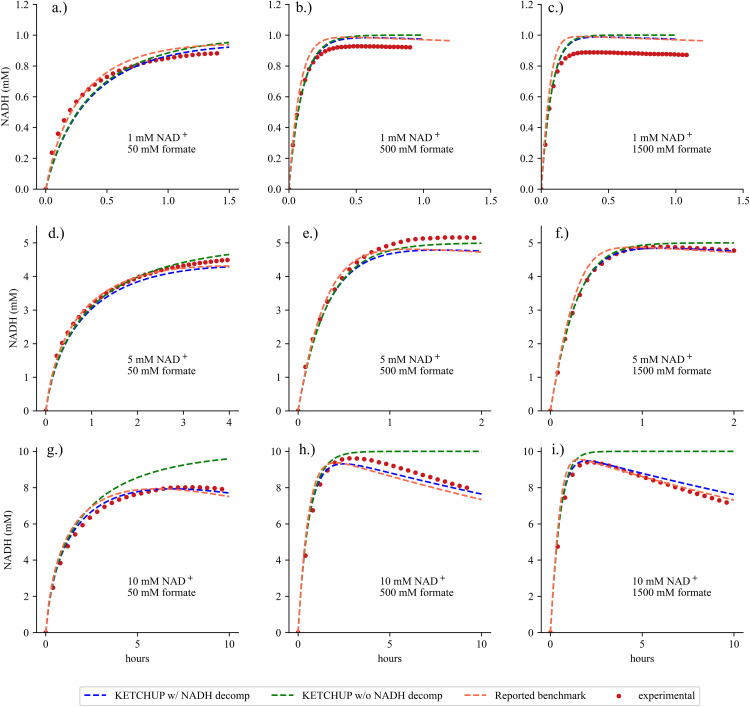
Prediction of NADH concentration over time between experimental data used in parameterization (red dots), simulation using KETCHUP-determined parameters with (dashed blue line) and without (dashed green line) NADH decomposition reaction, and previously reported fitted kinetic parameters (dashed orange line).

Three distinct time-course dataset series generated in this study (*i.e.,* two formate dehydrogenase, FDH and one 2,3-butanediol dehydrogenase, BDH) were curated and used for the parameterization of two distinct kinetic models. Both models were constructed following their reaction mechanism depicted in Fig 1. To first assess KETCHUP capability of fitting time-course data, we parameterized a FDH model with benchmarked data (dataset series A1) and compare kinetic parameters found with their respective reported values [80]. Similar parameter fits were achieved compared to the benchmarked kinetic parameters (See Fig 2). Next, we proceeded with the parameterization of datasets generated in this study for the FDH (dataset series B1 and B2) and BDH (dataset series Z1) systems with and without time-lag adjustments. These adjustments were made to help reconcile reaction start time and reported measurement times. We observed that time-lag adjustments only improve fitting of the datasets by 15%. Finally, we examine KETCHUP’s simulation capability of using single-enzyme identified kinetic parameters to simulate a binary system in a fed-batch system. Results indicate that single-enzyme fitted kinetic parameters can be combined and used in larger kinetic models for prediction of metabolite profiles (see Fig 4).

**Fig 3:**
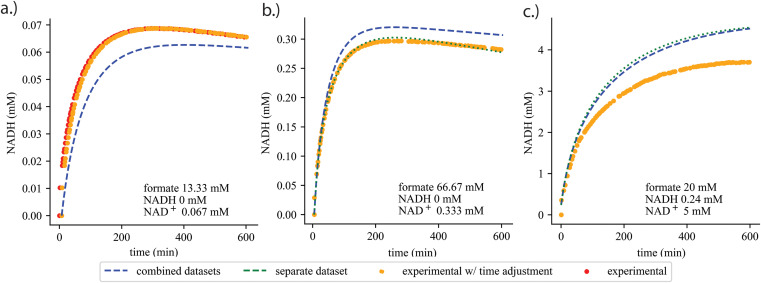
Prediction of NADH concentration for FDH over time between experimental (red dots), experimental with adjusted time delay (orange dots), simulation of separately parameterized dataset (dotted green line), and simulation of combined datasets (dashed blue line). Plots demonstrate **a)** improvement of fit (SSR) with time-delay parameter introduced, **b)** separately parameterized set verifying proposed mechanistic rate law can and **c)** cannot fit dataset. Initial metabolite concentrations are in plot text. Plots of prediction and simulation of all datasets for both FDH and BDH in Figs D–F in S4 File.

**Fig 4:**
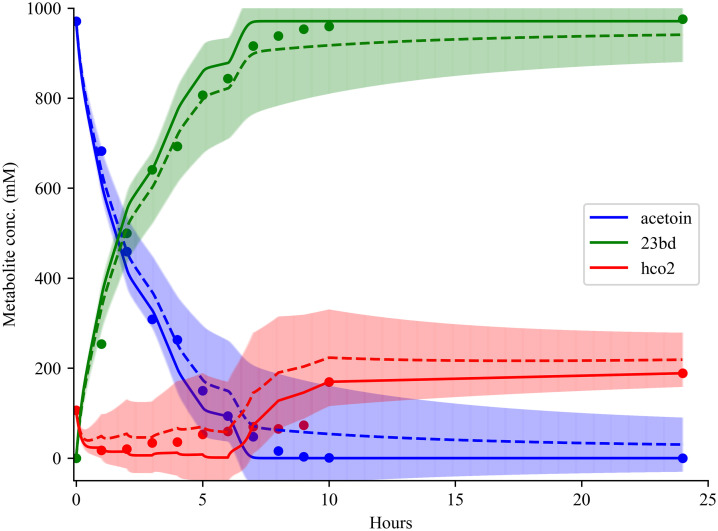
Prediction of Binary FDH-BDH system against experimental data (dotted points), best solutions simulation (solid lines) and mean and standard deviation of simulations with FDH and BDH alternative solutions (dashed lines). Piecewise simulation was performed using the best fitted solutions of single-enzyme parameterizations. Formate addition rate is extrapolated from experimental data points and assumed to be linear between each timepoints. Note that the introduction of adding formate periodically into the simulation results in a discontinuous metabolite simulation profile.

### Raw data filtering for parameterization

FDH time-course data with varying substrate, product, and enzyme concentrations were collected from a previous study [80] including (Dataset series A1) and two separate experiments (Datasets series B1 and B2). Due to unavailability of tabulated data from the literature study, Dataset series A1’s data points were extracted from the plot figure provided in the study using WebPlotDigitizer (automeris.io). BDH time course data with varying substrate, product, and enzyme concentrations were collected via experiments (Dataset series Z1). Experimental set-up and measurements are described in methods.

The collected raw time-course NADH concentration data were filtered by calculating a moving average of 10 datapoints per time point to smoothen out measurement noise. The data is then systematically thinned by using the Ramer-Douglas-Peucker (RDP) algorithm [81] to reduce the number of datapoints in a curve and consequently alleviate computational burden during parameterization with minimal loss of information. The threshold for the RDP algorithm for each dataset in each series is iteratively determined such that the resulting filtering would provide between 95–105 datapoints per dataset. This helps to normalize the error calculation for the objective function during fitting (see methods). Each raw data and corresponding filtered data were plotted (See Figs A–C in S4 File) with selected datasets being removed from further analysis if too noisy or exhibiting obviously erroneous trends (see S1 Text for exclusion criteria) following consultation with the data generation team. In summary, 13, 29, and 34 datasets out of 32, 88, and 134 were filtered from Dataset series B1, B2, and Z1, respectively. The initial RDP-filtered dataset series are in S8 Data–S11 Data while resulting curated dataset series used for parameterization can be found at https://github.com/maranasgroup/KETCHUP.

### Model construction

The FDH model was parameterized using a published simplified Michaelis-Menten style mechanistic rate law [80] (see Equation 1) where v is the reaction rate, V_max_ is maximal reaction rate, and A, B, and Q represent metabolite NAD^+^, formate, and NADH, respectively. Parameters K_M_ and K_I_ denote Michaelis-Menten and inhibitor constants, respectively. To parameterize varying enzyme concentrations V_max_ is set to be equal to a turnover number k_cat_ times the enzyme concentration E^FDH^. The BDH model was parameterized using a convenience kinetic mechanistic rate law [82] (see Equation 2), where $k_{cat}^{f}$ and $k_{cat}^{r}$ is the forward and reverse turnover number, respectively, S, P, and E^BDH^ representing metabolite acetoin and 2,3-butanediol and enzyme 2,3-butanediol dehydrogenase, respectively. To help constrain the reversible BDH, a thermodynamic constraint was introduced which required the turnover constants to comply with Haldane’s relationship (Equation 3) [83]. The standard Gibbs free energy, ΔG∘, was estimated to be -22.5 ± 4.1 kJ/mol using eQuilibrator 3.0 [84].

*Equation 1: Mechanistic kinetic rate law describing FDH presented by* [80].


$$v(FDH)=\frac{k_{cat}\;[A][B]}{K_{I}^{A}K_{M}^{B}+K_{M}^{B}[A]+K_{M}^{A}[B]+[A\;][B]+\frac{K_{I}^{A}K_{M}^{B}}{K_{I}^{Q}}[Q]+\frac{K_{M}^{A}}{K_{I}^{Q}}[B][Q]}[E^{FDH}]$$

*Equation 2: BDH rate law represented in convenience kinetics presented by* [82].


$$v(BDH)=\frac{\frac{k_{cat}^{f}[S][Q]}{K_{M}^{S}K_{M}^{Q}}-\frac{k_{cat}^{r}[P][A]}{K_{M}^{P}K_{M}^{A}}}{\left(\text{1}+\frac{\left[S\right]}{K_{M}^{S}}\right)\left(\text{1}+\frac{\left[Q\right]}{K_{M}^{Q}}\right)+\left(\text{1}+\frac{\left[P\right]}{K_{M}^{P}}\right)\left(\text{1}+\frac{\left[A\right]}{K_{M}^{A}}\right)\;-\text{1}}[E^{BDH}]$$

*Equation 3: Haldane relationship for BDH, where R is the Boltzmann’s gas constant and T is temperature in Kelvins:*


$$K_{eq}=\frac{K_{cat}^{f}K_{M}^{P}K_{M}^{A}}{K_{cat}^{r}K_{M}^{S}K_{M}^{Q}}=e^{-\frac{\Delta G\circ }{RT}}\;$$

Both kinetic models include an irreversible non-enzymatic decomposition reaction [85] for cofactor NADH for a more accurate description of the reaction dynamics over a long period of time (See Equation 4) where k_dQ_ is the NADH decomposition rate constant.

*Equation 4: NADH decomposition is assumed to follow first-order kinetics as presented in* [80].


$$v\left(NADH_{decomp}\right)=-k_{dQ}[Q]$$

### Comparison of kinetic parameters against a benchmark study

To benchmark KETCHUP’s extension towards parameterizing time-course data, we fitted the FDH kinetic model, containing reaction rate law for FDH and an irreversible first order NADH decomposition, with nine published datasets with varying initial NADH and formate concentrations [80]. Due to lack of tabulated datasets, the dataset series A1 was digitized to contain between 24–37 evenly spaced datapoints for each set. To sample across the solution space, 100 randomly initialized multi-starts were performed. 79% of the solutions reached convergence with an average solve time of 9.82 seconds. The best solution yielded a sum of squared residuals (SSR) value of 1.44 mM^2^ (similar to the SSR found while using benchmarked kinetic parameters with SSR of 1.49 mM^2^). The kinetic parameters for both KETCHUP-parameterized, MATLAB-parameterized, and benchmarked models are listed in Table 1. Mean and standard deviations for KETCHUP-based kinetic parameters were evaluated using the models yielding the best SSR and alternative models within 10% of the best model’s SSR as done in a previous study [86]. Except for inhibitor constant for NAD^+^, all kinetic parameters found with KETCHUP are within the same order of magnitude as benchmarked values. This discrepancy between KETCHUP-based and benchmarked kinetic parameters demonstrates that KETCHUP can find multiple kinetic parameters that can equally fit the data well and results in a larger standard deviation for some parameter values. Furthermore, there are parameters (*e.g.,* k_dQ_ and k_cat_) that are well resolved and within a fold of the benchmark values. A slight systematic overprediction of NADH for simulations with initial concentration of 1 mM NAD^+^ results from the objective function prioritizing minimizing the error deviation between predicted and experimental values for higher NADH concentrations. This prioritization consequently results in overprediction for the low NADH concentrations. It is important to also note that the inclusion of NADH degradation reaction better explains the datasets measured over longer periods of time [80] (See Fig 2g–2i) as the SSR increases to 3.73 mM^2^ if NADH degradation is unaccounted for in the simulation.

**Table 1:** Average kinetic parameters found during parameterization of FDH and their published value. Fitted kinetic parameters mean and standard deviations were evaluated using models yielding the best SSR and alternative models within 10% of the best model’s SSR.

Kinetic parameter	Fitted values (KETCHUP)	Fitted values (MATLAB)	Benchmarked values^a^	Log_2_ fold change KETCHUP (MATLAB)
$k_{dQ}$ (h^-1^)	2.9 × 10^-2^ ± 3 × 10^-12^	2.9 × 10^-2^ ± 2 × 10^-5^	3.45 × 10^-2^	-0.24 (-0.25)
$K_{I}^{A}$ (mM)	1.7 ± 8 × 10^-8^	6.3 ± 4.0	78.14 ± 0.08	-5.57 (-3.63)
$K_{i}^{Q}$ (mM)	1.5 × 10^-1^ ± 6 × 10^-9^	3.0 × 10^-1^ ± 1.3 × 10^-1^	1.18 × 10^-1^ ± 4.0 × 10^-5^	0.33 (1.37)
$K_{M}^{B}$ (mM)	2.2 × 10^1^ ± 1 × 10^-7^	1.4 × 10^1 ^± 5.5	4.72 × 10^-1 ^± 7 × 10^-3^	5.53 (4.90)
$K_{M}^{A}$ (mM)	6.6 × 10^-2^ ± 3 × 10^-9^	1.0 × 10^-1^ ± 4.5 × 10^-2^	3.84 × 10^-2^ ± 9 × 10^-5^	0.76 (1.38)
k_cat_ (s^-1^)	0.19 ± 2 × 10^-6^	0.18 ± 1.2 × 10^-2^	0.18	0.12 (0.02)

^a^Schmidt et al. 2010.

KETCHUP’s parameterization computation time performance for this dataset series is comparable to MATLAB’s [87] lsqnonlin function coupled with ode45 and has slightly better SSR (1.44 mM^2^ vs 1.49 mM^2^ and 8.49 vs 9.27 seconds, respectively). Kinetic parameter values found between KETCHUP and MATLAB are within two-fold from each other, indicating similar performance on the same datasets. Possible discrepancies between these kinetic values compared to the benchmarked values lies in the dataset and possibly reported variance of measurements (*i.e.,* KETCHUP and MATLAB fitting were performed with WebPlotDigitizer. These metrics show that despite KETCHUP’s original development for the parameterization of large-scale models using steady-state dataset, KETCHUP performs equally well to existing tools (*i.e.,* MATLAB) for time-course datasets (note that reported values in [80] were found using gPROMS (Version 3.0.2, Process System Enterprise Ltd., London, UK)).

### Fitting experimental datasets and time-lag considerations

Two FDH and one BDH kinetic models were parameterized with the generated herein dataset series B1, B2, and Z1, respectively. All kinetic models were first parameterized with their corresponding NADH decomposition standards to determine a range of values for $k_{dQ}$ per dataset series before parameterization of other parameters (*i.e.,* [1.50,3.50]×10^-4^ min^-1^, [1.11,1.77]×10^-4^ min^-1^, [1.286,1.287]×10^-3^ min^-1^ for dataset series B1, B2, and Z1, respectively). Each kinetic model was parameterized while constraining $k_{dQ}$ to their predetermined range with 500 randomly initialized multi-starts. Performance metrics are listed in Table 3. Note that $k_{dQ}$ for FDH experiments is one order of magnitude less than that for BDH experiments resulting from different experimental set-up (*i.e.,* assay buffer).

Initial observation of predicted and experimental NADH time-course shows poor fitting where slight x-axis (time) shifts in experimental NADH concentrations occur (see Fig 3a). This shift is a systematic time-lag that was introduced into the dataset because there is an unaccounted time-lapse between the reaction start time (*i.e.,* when the enzyme is mixed in with the metabolites) and the spectrophotometer measurement time. The resulting poor simulation fitting results from a mismatch of attempting to fit the datasets while fixing their initial measured concentrations. To correct this, KETCHUP’s utility function proactively searches via a bracketing search method to recommend an optimal time-lag that minimizes the SSR for each dataset while constraining the initial metabolite concentrations. Each dataset in series B1, B2, and Z1 are separately parameterized to find a corresponding time-lag parameter. Then each dataset series is re-parameterized with time-lag adjustments (see Figs D–F in S4 File). The implementation of time-lag adjustments was able to slightly improve SSR and solutions converged (See Table 3). However, time-lag parameters for each dataset in any given dataset series are distinct from one another indicating possible error in experimental setup resulting in uneven lags across datasets on top of possible measurement noise error.

Time-lag adjustments also slightly improved kinetic parameter resolution (See Table 2). We determined kinetic parameter resolution by observing the coefficient of variation (CoV) which is the ratio of the standard deviation to the mean and used to identify the degree of variation in kinetic parameters [88]. There is substantial improvement for the only NADH involved kinetic parameter for both FDH (*i.e.,* $K_{I}^{Q}$ CoV of 1.12 vs 2.19 and 0.13 vs 1.64 for dataset series B1 and B2, respectively) and BDH (*i.e.,* $K_{M}^{Q}$ CoV of 1.08 vs 1.94), demonstrating the importance of time-delay adjustments for NADH profiles. We note that time-delay adjustment was unable to resolve non-NADH related kinetic parameters found using Dataset B2 and Z1. This implies that although time-lag adjustments can help improve parameter fitting for the measured metabolite (SSR comparison, see Table 3), other kinetic parameters require their corresponding metabolite profile to improve resolution. However, we further verified the significance of the improvement in SSR of the additional time-delay parameter with Bartlett’s χ^2^-test [89] with an α-value of 0.001 (See S2 Text for calculations). Overall, this improvement in kinetic parameters related to the measured metabolite highlights not only the importance of having well-aligned time-course data but also the inclusion of different metabolite data as well.

**Table 2:** Mean, standard deviation (std), and coefficient of variation (CoV) of kinetic parameters found for dataset series B1, B2, and Z1.

Parameter name^a^	With time delay		Without time delay		% CoV improvement
	Mean ± std	CoV	Mean ± std	CoV	
Dataset series B1 – Formate dehydrogenase					
$K_{I}^{A}$ (mM)	(4 ± 11) ×10^3^	2.96	(8 ± 31) ×10^2^	3.83	22.7
$K_{I}^{Q}$ (mM)	(2.0 ± 2.2) ×10^4^	1.12	(7 ± 16) ×10^3^	2.19	48.9
$K_{M}^{B}$ (mM)	(4 ± 12) ×10^3^	2.86	(2.0 ± 8.4) ×10^3^	4.28	33.2
$K_{M}^{A}$ (mM)	(2.4 ± 3.3) ×10^4^	1.36	(9 ± 21) ×10^3^	2.4	43.3
$k_{cat}$ (s^-1^)	(5 ± 18) ×10^1^	3.85	(1 ± 11) ×10^1^	8.60	55.2
Dataset series B2 – Formate dehydrogenase					
$K_{I}^{A}$ (mM)	(6.40 ± 5.88) ×10^-1^	0.90	(4.4 ± 3.9) ×10^4^	0.89	-1.1
$K_{I}^{Q}$ (mM)	(1.11 ± 0.15) ×10^-1^	0.13	(1.42 ± 0.23) ×10^-1^	1.64	92.1
$K_{M}^{B}$ (mM)	(4 ± 23) ×10^2^	5.32	(1.7) ×10^1^	4.81	-10.6
$K_{M}^{A}$ (mM)	(2 ± 10) ×10^2^	5.24	(3 ± 16) ×10^2^	5.14	-1.9
$k_{cat}$ (s^-1^)	(7 ± 3.0) ×10^-1^	4.27	(6.5 ± 2.3)×10^2^	3.44	-24.1
Dataset series Z1 – 2,3-Butanediol dehydrogenase					
$K_{M}^{P}$ (mM)	(3.8 ± 8.5) ×10^1^	2.24	(5.0 ± 5.0) ×10^4^	1.0	-124
$K_{M}^{A}$ (mM)	(3.5 ± 3.1) ×10^4^	0.89	(5.6 ± 7.2) ×10^2^	1.28	30.5
$K_{M}^{S}$ (mM)	(0.6 ± 0.4) ×10^-1^	0.63	(3.9 ± 1.5) ×10^1^	0.38	-65.8
$K_{M}^{Q}$ (mM)	(1.6 ± 1.2) ×10^2^	0.40	1.7 ± 1.4	0.85	52.9
$k_{cat}^{f}$ (s^-1^)	(22.5 ± 1.6) ×10^3^	0.07	(4.3 ± 4.3) ×10^4^	1.0	93
$k_{cat}^{r}$ (s^-1^)	(3.7 ± 3.3) ×10^-1^	0.89	1.7 ± 2.2	1.26	29.4

a $K_{M}$, $K_{I}$, and $k_{cat}$ represents Michaelis-Menten, inhibitor, and turnover constants, respectively. A, B, S, P, and Q represent NAD^+^, formate, acetoin, 2,3-butanediol, and NADH, respectively.

**Table 3:** Performance metrics for parameterization of dataset series B1, B2, and Z1.

		With time delay			Without time delay		
Dataset Series (Enzyme)	# of datasets in series (DF)	Best SSR^a^ (mM^2^)	% solutions converged	Average solve time (seconds)	Best SSR (mM^2^)	% solutions converged	Average solve time (seconds)
B1 (FDH)	19 (7)	1.94	95.6	138	2.31	81	149
B2 (FDH)	59 (7)	162.70	63.6	674	194.61	16.4	767
Z1 (BDH)	78 (8)	45.93	12.6	533	46.38	5	533

^a^SSR value for each kinetic model solution falls within the $\chi^{\text{2}}$ statistic for a p-value of 0.05 with their corresponding degrees of freedom (DF).

After parameterization of kinetic models, we compare the fitting datasets to their simulation from *(a)* single-dataset and *(b)* full dataset series parameterized solution. A few simulations from scenario *(a)* show substantial deviation from the fitted datapoints (See example in Fig 3b). Normally, we would only expect that deviations occur in scenario (*b*) because the kinetic model is attempting to explain a variety of metabolite and enzyme concentrations with only one set of kinetic parameters. This discrepancy in scenario *(a)* likely results from either *(i)* poor selection of rate law representing the enzyme, *(ii)* lacking information on substrate or product inhibition, or (*iii)* noise in experimental measurements.

### Simulation of fed-batch binary system with single-enzyme kinetic parameters

To validate the assumption that kinetic parameters found in single-enzyme parameterizations can be used in multi-enzyme systems, an FDH-BDH binary system was simulated and compared against experimental results [90]. The initial experimental conditions are set to 971 mM of acetoin, 1 mM of NADH and 104 mM of formate measured with HPLC based on communications with the authors. Because the experiment implemented pH control (*i.e.,* addition of formic acid) and removed volume for samples, experimental data used for comparison were re-normalized to millimolar units after accounting for volume change. Simulation of the binary system was performed in piecewise fashion with each time-step matching the duration (*i.e.,* 60 minutes between sampling) between sampling timepoints with the previous end metabolite concentrations being reset as the initial condition in the next time-step. This piecewise simulation allows for flexible adjustment of formic acid addition rates to the simulation. Due to lack of pH control information, formate addition rate is assumed to be a linear between experimental datapoints (*e.g.*, a formate addition rate of 4.63 mM/minutes was assumed between timepoints 0 and 60 minutes). Note that the varying formate addition rates result in “non-smooth” metabolite profiles. The simulation was performed using the best solution from both enzymes (See Fig 4, solid line) and with combinations of alternative solutions near the best solution (See Fig 4, dashed lines and error bars). The best solutions and the alternative solutions were able to closely replicate the experimental metabolite profile (See Fig 4) and demonstrate ~100% and ~84% theoretical yield of 2,3-butanediol like the experimental results, respectively. Overall, this result demonstrates the feasibility of first resolving kinetic parameters for single enzymes and subsequently using the determined values to simulate multi-enzyme reaction cascades.

## Discussion

The parameterization of kinetic models for cell-free systems, specifically single-enzymes, offers potential in exploration and identification of rate laws and regulation to help better characterize each enzyme [59]. However, the parameterization of single-enzymes requires metabolite time-course data of varying initial concentrations. Herein, KETCHUP demonstrated fitting of single-enzyme datasets with time-lag correction and a resulting binary enzyme system, using single-enzyme fitted kinetic parameters, can closely simulate and replicate experimental metabolite profiles. These results demonstrate opportunities to efficiently characterize single enzymes for the systematic piecemeal construction of larger kinetic models that simulate cascade systems (*e.g.,* combining four separate kinetic and enzyme information to create a quad-enzyme system converting pyruvate to 2,3-butanediol [90]).

In this study, we first benchmarked KETCHUP’s performance and kinetic parameter estimations with a previously studied enzyme and characterized mechanistic rate law, formate dehydrogenase from *C. boidinii*. The best parameterized model (*i.e.,* model yielding the lowest SSR) was able to adequately recapitulate the fitted data. We observed a slight systematic overprediction in NADH time-course for lower initial NAD^+^ concentrations resulting from the objective of prioritizing kinetic parameters to better fit those with high NADH concentrations. For example, if all datapoints (*i.e.,* NADH concentrations) are weighed equally, time courses with higher NADH concentrations are valued more so when minimizing the SSR (see Equation 5), resulting in kinetic parameters fitting towards profiles with higher NADH concentrations. This overprediction characteristic can be adjusted to consider both low and high concentration NADH profiles during fitting by including weights to normalize the objective function. However, it is important to carefully consider weights for each datapoint in a way that does not bias the overall fit (*e.g.,* measurements for lower NADH concentrations may be within the range of experimental noise).

An important advantage purified enzyme cell-free systems has over *vivo* systems is the straightforward reaction environment that allows for selective metabolite testing (*i.e.,* addition/removal of known metabolites) for the observation of kinetic rates and allosteric regulations. Several factors can improve the resolution of kinetic parameters such as inclusion of different metabolite profiles and proper timed measurements. For example, NADH can be readily measured via spectrophotometer but CO_2_ (*i.e.,* product of FDH) cannot be measured in 384 well-plates. For the latter factor, KETCHUP provides a method to correct time discrepancies in high-throughput measurements by actively searching for the most suitable time-lag parameter to best fit the datasets.

We parameterized FDH and BDH kinetic models capable of recapitulating most experimental datasets. In this effort, we found multiple kinetic parameters capable of fitting the experimental data. However, some of these parameters lack resolution and have a wider distribution than most. This wider distribution can be attributed to lack of metabolite profiles that can help fix certain kinetic parameters in the rate law. Expanding the experimental design to include multiple metabolite profiles would benefit kinetic parameter resolution. For example, the benchmarked FDH rate law used did not include any Michaelis-Menten constants related to product metabolites (*i.e.,* CO_2_ and NADH) assuming that CO_2_ is rapidly dispersed into gaseous phase which renders reaction FDH irreversible [80]. However, this assumption is only true under very specific experimental conditions. For example, in the presence of active pH control (as we used in our two-enzyme FDH-BDH cascade reaction), the addition of concentrated acid causes rapid CO_2_ evolution from the liquid phase. On the other hand, when pH is controlled via buffering, the conversion of CO_2_ between its various carbonate forms is highly reversible and can affect FDH reversibility rates [91] and cascade reactions involving FDH [90].

For BDH, there is no published mechanistic rate law available, so convenience kinetics [82] was used as a starting point because the rate law is capable of capturing enzyme saturation effects, reaction stoichiometries, and allosteric regulations while only requiring a small number of parameters. Discrepancies in the fitting of BDH data likely result from the experimental set-up. Because BDH consumes NADH and there is a temperature discrepancy during the initial stages of the experiment provided in this study, NADH profiles display sigmoidal behavior where BDH activity gradually speeds up over time. This lag-phase hinders parameterization of BDH by obscuring the true initial NADH concentration for when enzyme assay reaches experimental temperature. By trimming the lag-phase out of the datasets and assuming no NADH consumption before the enzyme assay reaches temperature, KETCHUP was able to still successfully find kinetic parameters that can recapitulate most of the fitting datasets using the rate law defined with convenience kinetics. We note that although it is possible to better fit BDH data with another rate law and/or inclusion of inhibition information, convenience kinetics rate laws can be used as a decent starting point for exploratory purposes. Using the solutions from both FDH and BDH kinetic models, an FDH-BDH binary system kinetic model was developed, simulated, and evaluated against experimental data. This capability demonstrates the potential of hierarchically building kinetic models by first parameterizing one enzyme system at a time and subsequently integrating all parameterized enzyme kinetics into a single model to simulate the multi-reaction cascade.

Overall, cell-free systems are ideally positioned for rational and systematic enzyme characterizations for both rate laws and allostery exploration. Their high throughput combined with algorithmic methods to filter out noise allows for rapid parameterization of kinetic parameters. The extension introduced in this study serves as a pilot for parameterizing time-course data with KETCHUP, an open-source framework that was originally developed for scalable parameterization of large-scale kinetic systems using steady-state datasets with flexible objective function and customizable rate laws. This extension of KETCHUP enhances the design-test-build-learn cycle by demonstrating an automatable workflow that can speed-up decisions in experimental design (*e.g.,* which metabolite profiles to capture or initial metabolite concentrations). These features allow for straightforward implementation of user-defined searches (*e.g.,* finding the lowest set of kinetic parameters while minimizing SSR) and specific kinetic rate laws (*i.e.,* user-defined FDH reaction in this study). This tool’s adaptability towards user selections facilitates the semi-automatic construction and parameterization of customizable kinetic models compared to existing options such as MATLAB, which requires licensing or COPASI defaults kinetic rate-law selections to either mass action kinetics or simple Michaelis-Menten equations and prevents user-defined rate law. Development of these kinetic models to characterize enzyme using cell-free systems serves as a platform for the development of detailed large-scale kinetic models and application of these models towards engineering *in vivo* systems.

## Materials and methods

### Data collection

In this study, we collected NADH time-course data for *C. boidinii* FDH enzyme purchased from Sigma (Roche) (*i.e.,* Dataset series B1 and B2) and purified BDH protein sequenced from *S. marcescen* (*i.e.,* Dataset series Z1). Dataset series vary by initial substrate and enzyme concentrations ranges.

Individual enzyme activity measurements were performed in a 384 well plate in BioTek plate reader at 37^o^C. Activity was determined based on changes in the concentration of NADH, determined spectrophotometrically at 340 nm. In some cases, additional measurements at 390 nm and 400 nm were used to extend the dynamic range of measurements. NADH concentrations (in mM) are provided for Datasets series B1, B2, and Z1 (See S1 Data–S7 Data). The absorption values were converted to NADH concentration using the following extinction coefficients: 6.220 mM/Abs_340_/cm, 0.4276 mM/Abs_390_/cm, 0.1205 mM/Abs_400_/cm. The Abs_340_ extinction coefficient is a well-established constant [92]. The values at 390 and 400 nm were determined empirically. For a 60 µl reaction in a 384 well plate, the pathlength was 0.623 cm.

### Purification of BDH protein

Purification of the BDH protein has been described in detail previously [90]. Briefly, *E. coli* BL21 DE3 harboring 6x tagged was cultured overnight in LB medium in the presence of ampicillin (100 µg/mL). The secondary culture was started using 1% of the saturated overnight culture and induced using 0.3 mM IPTG at OD_600_ of ~ 0.4 after cultivation at 37 °C. Post induction, cells were harvested after 16 h cultivation at 18 °C and stored in -80 °C overnight. Pellets were resuspended in lysis buffer consisting of 20 mM tris-HCl pH 7.5, 500 mM NaCl, 5 mM imidazole, 1 mg/mL lysozyme and 1mM phenylmethylsulfonyl fluoride. Cells were lysed using microtip sonicator. After centrifugation of lysate at 9,000*g* (1 h and 4 °C), the supernatant was filtered (0.45 µm) and purified via Ni-nitrilotriacetic acid (Ni-NTA) metal affinity chromatography. The components of wash buffer were 20 mM tris-HCl pH 7.5, 500 mM NaCl and 15 mM imidazole. Protein elution was performed by buffer consisting of 20 mM tris-HCl pH 7.5, 500 mM NaCl and 650 mM imidazole. The purified protein was dialyzed in 100 mM sodium phosphate buffer and purity checked on SDS-PAGE gel. The concentration of BDH was determined by bicinchoninic acid (BCA) protein assay kit (G-Biosciences, MO, USA).

### Time-lag adjustments

Dynamic data often needs to be corrected for a time-lag that occurs due to the difference between when the assay is started (by addition of the starting reagent) and when the data collection starts. This time-lag represents the amount of time required to add and mix the starting reagent, apply the sealing film (if used to prevent evaporation), load the plate into the plate reader instrument, and initiate the data collection. Typical values range from 10 to 60 seconds.

To identify the correct time lag (in seconds) for each dataset, a bracketing search method is used to iteratively search for an optimal time lag time parameter that minimizes the SSR of each dataset (See below). After the level of all time lags have been identified for each dataset, the value of each time lag parameter is fixed for all subsequent simulations. A kinetic model is parameterized using the time lags to improve fit and resolve parameters estimated.

An initial guess of time lag ($t^{\text{0}}$) is first predicted by extrapolating the measured NADH concentration back to its initial value by fitting the data points to a cubic polynomial and then identifying the root value closest to zero (for FDH) or [NADH_init_] for BDH. A cubic polynomial was the lowest degree polynomial found to fit the data well without any substantial deviation from the datasets (*i.e.,* SSR < 1E-4). This fitting is done using the NumPy package [93] and functions polyfit and roots.

STEP 1: Initialize counter k = 0, time lag search interval step size $i^{\text{0}}=t^{\text{0}}$, stop criteria ϵ=0.001.
STEP 2: Set time lag search interval [$t^{k}-i^{k},\;t^{k}+i^{k}]$.
STEP 3: Divide search interval into ten evenly spaced search times G with step size $s^{\text{0}}$ such that,

$$G=\{t^{k}-i^{k},t^{k}-i^{k}+s^{\text{0}},\;t^{k}-i^{k}+\text{2}s^{\text{0}},\ldots ,t^{k}+i^{k}\}$$

STEP 4: For g ϵ G, run KETCHUP with 20 multi-starts and record lowest SSR $r_{g}^{k}$ and corresponding time lag $t_{g}^{k}$.
STEP 5: If lowest SSR lower than best SSR, update best SSR $r^{best}$ and corresponding time lag value $t^{best}$.

$$If\;r_{g}^{k}<r^{best}:r^{best}:=r_{g}^{k}\;and\;t^{best}:=t_{g}^{k}$$

STEP 6: Update time lag search interval step size.

$$t^{k+\text{1}}:=t^{best}\;,\;i^{k+\text{1}}:=s^{\text{0}}$$

STEP 7: Check time interval step size.

$$If\;i^{k}<\epsilon :STOP\;else\;k\;:=k+\text{1}$$

### Model parameterization using time-course data

Because KETCHUP was originally developed to fit steady-state data, the Pyomo differential algebraic equation (DAE) package [94] was used to discretize the rate laws which are in the form of ordinary differential equations (ODE). This is done by first declaring metabolite concentrations (*i.e.,* NADH) as a derivative variable (*i.e.,* Pyomo DAE DerivativeVar component) and time as a continuous set (*i.e.,* Pyomo DAE ContinuousSet component). Once the rate law is set to their respective ODE, Pyomo DAE discretizes the ODE using the Implicit Euler method (*i.e.,* default setting of Pyomo DAE discretizer) with the number of finite elements equal the number of time-points for the respective dataset. Pyomo is then used to formulate the remaining stoichiometric and feasibility (*e.g.,* non-negative kinetic parameters and concentrations) constraints. The objective function is set to minimize the SSR between predicted and fitted data with an equal weighting of one for each data point (See Equation 5). The full formulation is provided in S3 Text. Parameterization is then carried out with the nonlinear programming solver, Interior Point Optimizer (IPOPT [95]) with default threshold settings (See [79] for full details on IPOPT implementation in KETCHUP). Simulation of metabolite concentrations, for single and binary enzymes, are implemented using Pyomo DAE’s built-in simulator package which utilizes the CasADi framework [96] which uses the Implicit Differential-Algebraic Solver (IDA) [97] to solve the differential-algebraic equations defined by the rate laws. Mean and standard deviations for KETCHUP-based kinetic parameters were evaluated using the models yielding the best SSR and alternative models within 10% of the best model’s SSR.

*Equation 5: Sum of squared residual (SSR) for minimizing objective function used in the parameterization of kinetic models with time-course data, where t is a discrete time point, y is the measured experimental (meas) and model predicted (pred) values, and k is the index of dataset used during parameterization for all datasets K.*


$$SSR=\sum_{k\in K}\frac{{\left(y_{k}^{meas}(t)-y_{k}^{pred}(t)\right)}^{\text{2}}}{\text{1}}$$

### Computational implementation

KETCHUP is programmed in Python v3.8.5 and makes use of library packages provided through Anaconda. Formulation of equations and constraints are done through Pyomo v6.4.0. Parameterization and solving of equations are done with IPOPT v3.14.0, which interfaces with Pyomo, was recompiled from source code using GNU compiler v8.3.1 [98] to include linear solver ma97 [99] from the Harwell Subroutine Library [100]. Computations were performed on dual 10- or 12-core Xeon E5-2680 processors with InfiniBand using 1 core and 4 GB RAM running Red Hat Enterprise Linux Server release 7.9. KETCHUP uses COBRApy v0.25.0 [101] for reaction and metabolite parsing, Pandas v1.2.25 [102] for internal data structures and storing experimental data, and NumPy v1.23.1 [93] to randomize initial kinetic parameters. Converged kinetic model solutions can be exported to SBML [103] format using the libSBML package [104]. A generated graphical user interface is developed using Streamlit [105].

## Supporting information

S1 Data Dataset series B1. (XLSX)

S2 Data Dataset series B1 setup. (XLSX)

S3 Data Dataset series B2. (CSV)

S4 Data Dataset series B2 setup. (XLSX)

S5 Data Dataset series Z1 (setup and NADH decomposition included in file). (XLSX)

S6 Data NADH decomposition dataset for B1. (CSV)

S7 Data NADH decomposition dataset for B2. (CSV)

S8 Data Dataset series A1. (XLSX)

S9 Data Dataset series B1. (XLSX)

S10 Data Dataset series B2. (XLSX)

S11 Data Dataset series Z1. (XLSX)

S12 Data Validation Dataset for binary system. (XLSX)

S1 Text Data removed notes (notes on data removal for filtering process). (DOCX)

S2 Text Formulation of FDH and BDH and Bartlett’s test of significance for models. (DOCX)

S1 File RDP Data Thinning (BDH example). (IPYNB)

S2 File Simulation binary test (ipynb file for simulation of binary system). (IPYNB)

S3 File MATLAB code for parameterization of Dataset A1. (M)

S4 File Supplementary figures. Fig A. raw data fitting for Dataset B1. Fig B. raw data fitting for Dataset B2. Fig C. raw data fitting for Dataset Z1. Fig D. simulation of best solution for Dataset B1 with and without time-lag. Fig E. simulation of best solution for Dataset B2 with and without time-lag. Fig F. simulation of best solution for Dataset B1 with and without time-lag.
(DOCX)

S5 File FDH_optimal_results_18. (JSON)

S6 File FDH_optimal_results_25. (JSON)

S7 File FDH_optimal_results_32. (JSON)

S8 File FDH_optimal_results_38. (JSON)

S9 File FDH_optimal_results_61. (JSON)

S10 File FDH_optimal_results_132. (JSON)

S11 File FDH_optimal_results_192. (JSON)

S12 File FDH_optimal_results_291. (JSON)

S13 File BDH_optimal_results_101. (JSON)

S14 File BDH_optimal_results_212. (JSON)

S15 File BDH_optimal_results_302. (JSON)

S16 File BDH_optimal_results_478. (JSON)

S17 File KETCHUP_dynamic_yaml (run instructions and data at https://github.com/maranasgroup/KETCHUP). (PY)

S18 File Options_dynamic_BDH. (YML)

S19 File Options_dynamic_FDH. (YML)

## References

[1] Sheng J, Feng X. Metabolic engineering of yeast to produce fatty acid-derived biofuels: bottlenecks and solutions. Front Microbiol. 2015;6:554. doi: 10.3389/fmicb.2015.00554 26106371 PMC4459083

[2] Choi KR, Jiao S, Lee SY. Metabolic engineering strategies toward production of biofuels. Curr Opin Chem Biol. 2020;59:1–14. doi: 10.1016/j.cbpa.2020.02.009 32298980

[3] Joshi A, Verma KK, D Rajput V, Minkina T, Arora J. Recent advances in metabolic engineering of microorganisms for advancing lignocellulose-derived biofuels. Bioengineered. 2022;13(4):8135–63. doi: 10.1080/21655979.2022.2051856 35297313 PMC9161965

[4] Paddon CJ, Westfall PJ, Pitera DJ, Benjamin K, Fisher K, McPhee D, et al. High-level semi-synthetic production of the potent antimalarial artemisinin. Nature. 2013;496(7446):528–32. doi: 10.1038/nature12051 23575629

[5] Khosla C, Keasling JD. Metabolic engineering for drug discovery and development. Nat Rev Drug Discov. 2003;2(12):1019–25. doi: 10.1038/nrd1256 14654799

[6] Navale GR, Dharne MS, Shinde SS. Metabolic engineering and synthetic biology for isoprenoid production in Escherichia coli and Saccharomyces cerevisiae. Appl Microbiol Biotechnol. 2021;105(2):457–75. doi: 10.1007/s00253-020-11040-w 33394155

[7] Kwak S, Kim SR, Xu H, Zhang G-C, Lane S, Kim H, et al. Enhanced isoprenoid production from xylose by engineered Saccharomyces cerevisiae. Biotechnol Bioeng. 2017;114(11):2581–91. doi: 10.1002/bit.26369 28667762

[8] Kirby J, Dietzel KL, Wichmann G, Chan R, Antipov E, Moss N, et al. Engineering a functional 1-deoxy-D-xylulose 5-phosphate (DXP) pathway in Saccharomyces cerevisiae. Metab Eng. 2016;38:494–503. doi: 10.1016/j.ymben.2016.10.017 27989805 PMC5718835

[9] Yu A-Q, Pratomo Juwono NK, Foo JL, Leong SSJ, Chang MW. Metabolic engineering of Saccharomyces cerevisiae for the overproduction of short branched-chain fatty acids. Metab Eng. 2016;34:36–43. doi: 10.1016/j.ymben.2015.12.005 26721212

[10] Runguphan W, Keasling JD. Metabolic engineering of Saccharomyces cerevisiae for production of fatty acid-derived biofuels and chemicals. Metab Eng. 2014;21:103–13. doi: 10.1016/j.ymben.2013.07.003 23899824

[11] Novy V, Brunner B, Nidetzky B. L-Lactic acid production from glucose and xylose with engineered strains of Saccharomyces cerevisiae: aeration and carbon source influence yields and productivities. Microb Cell Fact. 2018;17(1):59. doi: 10.1186/s12934-018-0905-z 29642896 PMC5894196

[12] Xiberras J, Klein M, de Hulster E, Mans R, Nevoigt E. Engineering Saccharomyces cerevisiae for Succinic Acid Production From Glycerol and Carbon Dioxide. Front Bioeng Biotechnol. 2020;8:566. doi: 10.3389/fbioe.2020.00566 32671027 PMC7332542

[13] Naseri G, Koffas MAG. Application of combinatorial optimization strategies in synthetic biology. Nat Commun. 2020;11(1):2446. doi: 10.1038/s41467-020-16175-y 32415065 PMC7229011

[14] Tong T, Chen X, Hu G, Wang X-L, Liu G-Q, Liu L. Engineering microbial metabolic energy homeostasis for improved bioproduction. Biotechnol Adv. 2021;53:107841. doi: 10.1016/j.biotechadv.2021.107841 34610353

[15] Nielsen J, Keasling JD. Engineering Cellular Metabolism. Cell. 2016;164(6):1185–97. doi: 10.1016/j.cell.2016.02.004 26967285

[16] Vilkhovoy M, Dai D, Vadhin S, Adhikari A, Varner JD. Absolute Quantification of Cell-Free Protein Synthesis Metabolism by Reversed-Phase Liquid Chromatography-Mass Spectrometry. J Vis Exp. 2019;(152):10.3791/60329. doi: 10.3791/60329 31710042

[17] Borkowski O, Bricio C, Murgiano M, Rothschild-Mancinelli B, Stan G-B, Ellis T. Cell-free prediction of protein expression costs for growing cells. Nat Commun. 2018;9(1):1457. doi: 10.1038/s41467-018-03970-x 29654285 PMC5899134

[18] Hanson AD, McCarty DR, Henry CS, Xian X, Joshi J, Patterson JA, et al. The number of catalytic cycles in an enzyme’s lifetime and why it matters to metabolic engineering. Proc Natl Acad Sci U S A. 2021;118(13):e2023348118. doi: 10.1073/pnas.2023348118 33753504 PMC8020674

[19] Brookwell A, Oza JP, Caschera F. Biotechnology Applications of Cell-Free Expression Systems. Life (Basel). 2021;11(12):1367. doi: 10.3390/life11121367 34947898 PMC8705439

[20] Kohler R. The background to Eduard Buchner’s discovery of cell-free fermentation. J Hist Biol. 1971;4:35–61. doi: 10.1007/BF00356976 11609437

[21] Shimizu Y, Kanamori T, Ueda T. Protein synthesis by pure translation systems. Methods. 2005;36(3):299–304. doi: 10.1016/j.ymeth.2005.04.006 16076456

[22] Cui Y, Chen X, Wang Z, Lu Y. Cell-Free PURE System: Evolution and Achievements. Biodes Res. 2022;2022:9847014. doi: 10.34133/2022/9847014 37850137 PMC10521753

[23] Niwa T, Sasaki Y, Uemura E, Nakamura S, Akiyama M, Ando M, et al. Comprehensive study of liposome-assisted synthesis of membrane proteins using a reconstituted cell-free translation system. Sci Rep. 2015;5:18025. doi: 10.1038/srep18025 26667602 PMC4678891

[24] Murakami S, Matsumoto R, Kanamori T. Constructive approach for synthesis of a functional IgG using a reconstituted cell-free protein synthesis system. Sci Rep. 2019;9(1):671. doi: 10.1038/s41598-018-36691-8 30679500 PMC6345822

[25] Ueda T, Kanamori T, Ohashi H. Ribosome display with the PURE technology. Methods Mol Biol. 2010;607:219–25. doi: 10.1007/978-1-60327-331-2_18 20204860

[26] Hillebrecht JR, Chong S. A comparative study of protein synthesis in in vitro systems: from the prokaryotic reconstituted to the eukaryotic extract-based. BMC Biotechnol. 2008;8:58. doi: 10.1186/1472-6750-8-58 18664286 PMC2507708

[27] Zawada JF, Yin G, Steiner AR, Yang J, Naresh A, Roy SM, et al. Microscale to manufacturing scale-up of cell-free cytokine production--a new approach for shortening protein production development timelines. Biotechnol Bioeng. 2011;108(7):1570–8. doi: 10.1002/bit.23103 21337337 PMC3128707

[28] Voloshin AM, Swartz JR. Efficient and scalable method for scaling up cell free protein synthesis in batch mode. Biotechnol Bioeng. 2005;91(4):516–21. doi: 10.1002/bit.20528 15937883

[29] Gregorio NE, Levine MZ, Oza JP. A User’s Guide to Cell-Free Protein Synthesis. Methods Protoc. 2019;2(1):24. doi: 10.3390/mps2010024 31164605 PMC6481089

[30] Sawasaki T, Ogasawara T, Morishita R, Endo Y. A cell-free protein synthesis system for high-throughput proteomics. Available from: www.pnas.orgcgi; https://doi.org/10.1073/pnas.232580399 10.1073/pnas.232580399 PMC137474 12409616

[31] Swartz JR. Expanding biological applications using cell-free metabolic engineering: An overview. Metab Eng. 2018;50:156–72. doi: 10.1016/j.ymben.2018.09.011 30367967

[32] Thaore V, Tsourapas D, Shah N, Kontoravdi C. Techno-Economic Assessment of Cell-Free Synthesis of Monoclonal Antibodies Using CHO Cell Extracts. Processes. 2020;8(4):454. doi: 10.3390/pr8040454

[33] Lee K-H, Kwon Y-C, Yoo SJ, Kim D-M. Ribosomal synthesis and in situ isolation of peptide molecules in a cell-free translation system. Protein Expr Purif. 2010;71(1):16–20. doi: 10.1016/j.pep.2010.01.016 20100575

[34] Pardee K, Slomovic S, Nguyen PQ, Lee JW, Donghia N, Burrill D, et al. Portable, On-Demand Biomolecular Manufacturing. Cell. 2016;167(1):248-259.e12. doi: 10.1016/j.cell.2016.09.013 27662092

[35] Zhang Y-HP. Production of biofuels and biochemicals by in vitro synthetic biosystems: Opportunities and challenges. Biotechnol Adv. 2015;33(7):1467–83. doi: 10.1016/j.biotechadv.2014.10.009 25447781

[36] Bowie JU, Sherkhanov S, Korman TP, Valliere MA, Opgenorth PH, Liu H. Synthetic Biochemistry: The Bio-inspired Cell-Free Approach to Commodity Chemical Production. Trends Biotechnol. 2020;38(7):766–78. doi: 10.1016/j.tibtech.2019.12.024 31983463

[37] Korman TP, Opgenorth PH, Bowie JU. A synthetic biochemistry platform for cell free production of monoterpenes from glucose. Nat Commun. 2017;8:15526. doi: 10.1038/ncomms15526 28537253 PMC5458089

[38] Zhou J, Huang L, Lian J, Sheng J, Cai J, Xu Z. Reconstruction of the UDP-N-acetylglucosamine biosynthetic pathway in cell-free system. Biotechnol Lett. 2010;32(10):1481–6. doi: 10.1007/s10529-010-0315-8 20495944

[39] Orth JD, Thiele I, Palsson BØ. What is flux balance analysis? Nat Biotechnol. 2010;28(3):245–8. doi: 10.1038/nbt.1614 20212490 PMC3108565

[40] Costa RS, Hartmann A, Vinga S. Kinetic modeling of cell metabolism for microbial production. J Biotechnol. 2016;219:126–41. doi: 10.1016/j.jbiotec.2015.12.023 26724578

[41] Resat H, Petzold L, Pettigrew MF. Kinetic modeling of biological systems. Methods Mol Biol. 2009;541:311–35. doi: 10.1007/978-1-59745-243-4_14 19381542 PMC2877599

[42] Khodayari A, Zomorrodi AR, Liao JC, Maranas CD. A kinetic model of Escherichia coli core metabolism satisfying multiple sets of mutant flux data. Metab Eng. 2014;25:50–62. doi: 10.1016/j.ymben.2014.05.014 24928774

[43] St John PC, Bomble YJ. Approaches to Computational Strain Design in the Multiomics Era. Front Microbiol. 2019;10:597. doi: 10.3389/fmicb.2019.00597 31024467 PMC6461008

[44] Link H, Christodoulou D, Sauer U. Advancing metabolic models with kinetic information. Curr Opin Biotechnol. 2014;29:8–14. doi: 10.1016/j.copbio.2014.01.015 24534671

[45] Almquist J, Cvijovic M, Hatzimanikatis V, Nielsen J, Jirstrand M. Kinetic models in industrial biotechnology - Improving cell factory performance. Metab Eng. 2014;24:38–60. doi: 10.1016/j.ymben.2014.03.007 24747045

[46] Khodayari A, Chowdhury A, Maranas CD. Succinate Overproduction: A Case Study of Computational Strain Design Using a Comprehensive Escherichia coli Kinetic Model. Front Bioeng Biotechnol. 2015;2:76. doi: 10.3389/fbioe.2014.00076 25601910 PMC4283520

[47] Mishra S, Wang Z, Volk MJ, Zhao H. Design and application of a kinetic model of lipid metabolism in Saccharomyces cerevisiae. Metab Eng. 2023;75:12–8. doi: 10.1016/j.ymben.2022.11.003 36371031

[48] Hatzimanikatis V, Emmerling M, Sauer U, Bailey JE. Application of mathematical tools for metabolic design of microbial ethanol production. Biotechnol Bioeng. 1998;58(2–3):154–61. doi: 10.1002/(sici)1097-0290(19980420)58:2/3<154::aid-bit7>3.0.co;2-k 10191385

[49] Smallbone K, Messiha HL, Carroll KM, Winder CL, Malys N, Dunn WB, et al. A model of yeast glycolysis based on a consistent kinetic characterisation of all its enzymes. FEBS Lett. 2013;587(17):2832–41. doi: 10.1016/j.febslet.2013.06.043 23831062 PMC3764422

[50] Link H, Kochanowski K, Sauer U. Systematic identification of allosteric protein-metabolite interactions that control enzyme activity in vivo. Nat Biotechnol. 2013;31(4):357–61. doi: 10.1038/nbt.2489 23455438

[51] Saa PA, Nielsen LK. Construction of feasible and accurate kinetic models of metabolism: A Bayesian approach. Sci Rep. 2016;6:29635. doi: 10.1038/srep29635 27417285 PMC4945864

[52] Tran LM, Rizk ML, Liao JC. Ensemble modeling of metabolic networks. Biophys J. 2008;95(12):5606–17. doi: 10.1529/biophysj.108.135442 18820235 PMC2599852

[53] Miskovic L, Hatzimanikatis V. Production of biofuels and biochemicals: in need of an ORACLE. Trends Biotechnol. 2010;28(8):391–7. doi: 10.1016/j.tibtech.2010.05.003 20646768

[54] Cosgrove MS, Naylor C, Paludan S, Adams MJ, Levy HR. On the mechanism of the reaction catalyzed by glucose 6-phosphate dehydrogenase. Biochemistry. 1998;37(9):2759–67. doi: 10.1021/bi972069y 9485426

[55] Kochetov GA, Solovjeva ON. Structure and functioning mechanism of transketolase. Biochim Biophys Acta. 2014;1844(9):1608–18. doi: 10.1016/j.bbapap.2014.06.003 24929114

[56] Heijnen JJ, Verheijen PJT. Parameter identification of in vivo kinetic models: limitations and challenges. Biotechnol J. 2013;8(7):768–75. doi: 10.1002/biot.201300105 23813763

[57] Martin JP, Rasor BJ, DeBonis J, Karim AS, Jewett MC, Tyo KEJ, et al. A dynamic kinetic model captures cell-free metabolism for improved butanol production. Metab Eng. 2023;76:133–45. doi: 10.1016/j.ymben.2023.01.009 36724840

[58] Horvath N, Vilkhovoy M, Wayman JA, Calhoun K, Swartz J, Varner JD. Toward a genome scale sequence specific dynamic model of cell-free protein synthesis in Escherichia coli. Metab Eng Commun. 2019;10:e00113. doi: 10.1016/j.mec.2019.e00113 32280586 PMC7136494

[59] Laohakunakorn N. Cell-Free Systems: A Proving Ground for Rational Biodesign. Front Bioeng Biotechnol. 2020;8:788. doi: 10.3389/fbioe.2020.00788 32793570 PMC7393481

[60] Zielinski DC, Matos MRA, de Bree JE, Glass K, Sonnenschein N, Palsson BO. Bottom-up parameterization of enzyme rate constants: Reconciling inconsistent data. Metab Eng Commun. 2024;18:e00234. doi: 10.1016/j.mec.2024.e00234 38711578 PMC11070925

[61] Garenne D, Noireaux V. Cell-free transcription-translation: engineering biology from the nanometer to the millimeter scale. Curr Opin Biotechnol. 2019;58:19–27. doi: 10.1016/j.copbio.2018.10.007 30395952

[62] Takahashi MK, Hayes CA, Chappell J, Sun ZZ, Murray RM, Noireaux V, et al. Characterizing and prototyping genetic networks with cell-free transcription-translation reactions. Methods. 2015;86:60–72. doi: 10.1016/j.ymeth.2015.05.020 26022922

[63] Gopalakrishnan S, Dash S, Maranas C. K-FIT: An accelerated kinetic parameterization algorithm using steady-state fluxomic data. Metab Eng. 2020;61:197–205. doi: 10.1016/j.ymben.2020.03.001 32173504

[64] Matos MRA, Saa PA, Cowie N, Volkova S, de Leeuw M, Nielsen LK. GRASP: a computational platform for building kinetic models of cellular metabolism. Bioinform Adv. 2022;2(1):vbac066. doi: 10.1093/bioadv/vbac066 36699366 PMC9710608

[65] Jamshidi N, Palsson BØ. Mass action stoichiometric simulation models: incorporating kinetics and regulation into stoichiometric models. Biophys J. 2010;98(2):175–85. doi: 10.1016/j.bpj.2009.09.064 20338839 PMC2808481

[66] Palmisano A, Hoops S, Watson LT, Jones TC Jr, Tyson JJ, Shaffer CA. JigCell Run Manager (JC-RM): a tool for managing large sets of biochemical model parametrizations. BMC Syst Biol. 2015;9:95. doi: 10.1186/s12918-015-0237-0 26704692 PMC4690432

[67] Hoops S, Sahle S, Gauges R, Lee C, Pahle J, Simus N, et al. COPASI--a COmplex PAthway SImulator. Bioinformatics. 2006;22(24):3067–74. doi: 10.1093/bioinformatics/btl485 17032683

[68] Choi K, Medley JK, König M, Stocking K, Smith L, Gu S, et al. Tellurium: An extensible python-based modeling environment for systems and synthetic biology. Biosystems. 2018;171:74–9. doi: 10.1016/j.biosystems.2018.07.006 30053414 PMC6108935

[69] Weilandt DR, Salvy P, Masid M, Fengos G, Denhardt-Erikson R, Hosseini Z, et al. Symbolic kinetic models in python (SKiMpy): intuitive modeling of large-scale biological kinetic models. Bioinformatics. 2023;39(1):btac787. doi: 10.1093/bioinformatics/btac787 36495209 PMC9825757

[70] Hatzimanikatis V. Nonlinear metabolic control analysis. Metab Eng. 1999;1(1):75–87. doi: 10.1006/mben.1998.0108 10935756

[71] Saa P, Nielsen LK. A general framework for thermodynamically consistent parameterization and efficient sampling of enzymatic reactions. PLoS Comput Biol. 2015;11(4):e1004195. doi: 10.1371/journal.pcbi.1004195 25874556 PMC4397067

[72] Chen Y, Nielsen J. In vitro turnover numbers do not reflect in vivo activities of yeast enzymes. Proc Natl Acad Sci U S A. 2021;118(32):e2108391118. doi: 10.1073/pnas.2108391118 34341111 PMC8364156

[73] Dinh HV, Maranas CD. Evaluating proteome allocation of Saccharomyces cerevisiae phenotypes with resource balance analysis. Metab Eng. 2023;77:242–55. doi: 10.1016/j.ymben.2023.04.009 37080482

[74] Jewett MC, Swartz JR. Mimicking the Escherichia coli cytoplasmic environment activates long-lived and efficient cell-free protein synthesis. Biotechnol Bioeng. 2004;86(1):19–26. doi: 10.1002/bit.20026 15007837

[75] Jewett MC, Calhoun KA, Voloshin A, Wuu JJ, Swartz JR. An integrated cell-free metabolic platform for protein production and synthetic biology. Mol Syst Biol. 2008;4:220. doi: 10.1038/msb.2008.57 18854819 PMC2583083

[76] van Eunen K, Bouwman J, Daran-Lapujade P, Postmus J, Canelas AB, Mensonides FIC, et al. Measuring enzyme activities under standardized in vivo-like conditions for systems biology. FEBS J. 2010;277(3):749–60. doi: 10.1111/j.1742-4658.2009.07524.x 20067525

[77] Smallbone K, Simeonidis E, Swainston N, Mendes P. Towards a genome-scale kinetic model of cellular metabolism. BMC Syst Biol. 2010;4:6. doi: 10.1186/1752-0509-4-6 20109182 PMC2829494

[78] Fröhlich F, Kaltenbacher B, Theis FJ, Hasenauer J. Scalable Parameter Estimation for Genome-Scale Biochemical Reaction Networks. PLoS Comput Biol. 2017;13(1):e1005331. doi: 10.1371/journal.pcbi.1005331 28114351 PMC5256869

[79] Hu M, Suthers PF, Maranas CD. KETCHUP: Parameterizing of large-scale kinetic models using multiple datasets with different reference states. Metab Eng. 2024;82:123–33. doi: 10.1016/j.ymben.2024.02.002 38336004

[80] Schmidt T, Michalik C, Zavrel M, Spiess A, Marquardt W, Ansorge-Schumacher MB. Mechanistic model for prediction of formate dehydrogenase kinetics under industrially relevant conditions. Biotechnol Prog. 2010;26(1):73–8. doi: 10.1002/btpr.282 19830796

[81] Ramer U. An iterative procedure for the polygonal approximation of plane curves. Comput Graphics Image Process. 1972;1(3):244–56. doi: 10.1016/s0146-664x(72)80017-0

[82] Liebermeister W, Klipp E. Bringing metabolic networks to life: convenience rate law and thermodynamic constraints. Theor Biol Med Model. 2006;3:41. doi: 10.1186/1742-4682-3-41 17173669 PMC1781438

[83] Haldane JBS. Enzymes. London: Longmans, Grenn, and Co. reprinted by M.I.T. press. Cambridge, MA: reprinted by M.I.T press 1965; 1930.

[84] Beber ME, Gollub MG, Mozaffari D, Shebek KM, Flamholz AI, Milo R, et al. eQuilibrator 3.0: a database solution for thermodynamic constant estimation. Nucleic Acids Res. 2022;50(D1):D603–9. doi: 10.1093/nar/gkab1106 34850162 PMC8728285

[85] Chenault HK, Whitesides GM. Regeneration of nicotinamide cofactors for use in organic synthesis. Appl Biochem Biotechnol. 1987;14(2):147–97. doi: 10.1007/BF02798431 3304160

[86] Foster C, Boorla VS, Dash S, Gopalakrishnan S, Jacobson TB, Olson DG, et al. Assessing the impact of substrate-level enzyme regulations limiting ethanol titer in Clostridium thermocellum using a core kinetic model. Metab Eng. 2022;69:286–301. doi: 10.1016/j.ymben.2021.12.012 34982997

[87] The MathWorks Inc. MATLAB. Natick, Massachusetts, United States: The MathWorks Inc.; 2023.

[88] Reed GF, Lynn F, Meade BD. Use of coefficient of variation in assessing variability of quantitative assays. Clin Diagn Lab Immunol. 2002;9(6):1235–9. doi: 10.1128/cdli.9.6.1235-1239.2002 12414755 PMC130103

[89] Verheijen PJT. Model selection: An overview of practices in chemical engineering. Comput Aided Chem Eng. 2003;85–104. doi: 10.1016/s1570-7946(03)80071-8

[90] Jilani SB, Alahuhta M, Bomble YJ, Olson DG. Cell-Free Systems Biology: Characterizing Central Metabolism of Clostridium thermocellum with a Three-Enzyme Cascade Reaction. ACS Synth Biol. 2024;13(11):3587–99. doi: 10.1021/acssynbio.4c00405 39387698 PMC11574923

[91] Sato R, Amao Y. Can formate dehydrogenase from Candida boidinii catalytically reduce carbon dioxide, bicarbonate, or carbonate to formate? New J Chem. 2020;44(28):11922–6. doi: 10.1039/d0nj01183e

[92] Dawson RMC. Data for Biochemical Research. Clarendon Press; 1969. Available from: https://books.google.com/books?id=Okma5E8mPw0C

[93] Harris CR, Millman KJ, van der Walt SJ, Gommers R, Virtanen P, Cournapeau D, et al. Array programming with NumPy. Nature. 2020;585(7825):357–62. doi: 10.1038/s41586-020-2649-2 32939066 PMC7759461

[94] Nicholson B, Siirola JD, Watson J-P, Zavala VM, Biegler LT. pyomo.dae: a modeling and automatic discretization framework for optimization with differential and algebraic equations. Math Prog Comp. 2017;10(2):187–223. doi: 10.1007/s12532-017-0127-0

[95] Wächter A, Biegler LT. On the implementation of an interior-point filter line-search algorithm for large-scale nonlinear programming. Math Program. 2005;106(1):25–57. doi: 10.1007/s10107-004-0559-y

[96] Andersson JAE, Gillis J, Horn G, Rawlings JB, Diehl M. CasADi: a software framework for nonlinear optimization and optimal control. Math Prog Comp. 2018;11(1):1–36. doi: 10.1007/s12532-018-0139-4

[97] Hindmarsh AC, Brown PN, Grant KE, Lee SL, Serban R, Shumaker DE, et al. SUNDIALS: Suite of Nonlinear and Differential/Algebraic Equation Solvers. 2005.

[98] GNU Fortran Compiler Manuals. Available from: https://gcc.gnu.org/onlinedocs/gcc-8.3.0/gfortran/

[99] Hogg JD, Scott JA, Oxford H. HSL_MA97: a bit-compatible multifrontal code for sparse symmetric systems. Rutherford Appleton Laboratory Technical Reports. 2011. Available from: http://purl.org/net/epubs/manifestation/7236

[100] HSL. A collection of Fortran codes for large scale scientific computation.

[101] Ebrahim A, Lerman JA, Palsson BO, Hyduke DR. COBRApy: COnstraints-Based Reconstruction and Analysis for Python. BMC Syst Biol. 2013;7:74. doi: 10.1186/1752-0509-7-74 23927696 PMC3751080

[102] McKinney W. Data Structures for Statistical Computing in Python. In: Proceedings of the 9th Python in Science Conference. 2010;1: 56–61. doi: 10.25080/majora-92bf1922-00a

[103] Xu J. SBMLKinetics: a tool for annotation-independent classification of reaction kinetics for SBML models. BMC Bioinformatics. 2023;24(1):248. doi: 10.1186/s12859-023-05380-3 37312031 PMC10262532

[104] Bornstein BJ, Keating SM, Jouraku A, Hucka M. LibSBML: an API library for SBML. Bioinformatics. 2008;24(6):880–1. doi: 10.1093/bioinformatics/btn051 18252737 PMC2517632

[105] Snowflake Inc. Streamlit. 2023. Available from: https://github.com/streamlit/streamlit

