# Supplementary material for: Parameterization of cell-free systems with time-series data using KETCHUP
Source: PLoS Comput Biol. 2025 Nov 21;21(11):e1013724. doi: 10.1371/journal.pcbi.1013724 (PMC12637948; doi:10.1371/journal.pcbi.1013724)
Supplement: S1 Text — (DOCX) [file pcbi.1013724.s013.docx]

threshold greater than SSR 2E-04 means datasets cannot be explained properly with mechanistic rate law

this may be resulting from mismatched initial conditions in the parameterization against experimental measurements.

FDH Olson02 - Dataset series B1

C1 - flat line of data points, does not show any curve

C2 - flat line of data points, does not show any curve

D1 - flat line of data points, does not show any curve

D2 - flat line of data points, does not show any curve

G1 - data "curve" close to 90 degrees, with flat horizontal line, cannot be explained with mechanistic rate law

G2 - data "curve" close to 90 degrees, with flat horizontal line cannot be explained with mechanistic rate law

H2 - flat line of data points, does not show any curve

L1 - data "curve" close to 90 degrees, with flat horizontal line, cannot be explained with mechanistic rate law

L2 - data "curve" close to 90 degrees, with flat horizontal line, cannot be explained with mechanistic rate law

P1 - data "curve" close to 90 degrees, with flat horizontal line cannot be explained with mechanistic rate law

P2 - data "curve" close to 90 degrees, with flat horizontal line, cannot be explained with mechanistic rate law

M1 - noisy data points, multiple unexplained outliers, near straight diagonal line

M2 - noisy data points, multiple unexplained outliers, near straight diagonal line

FDH Olson03 - Dataset series B2

A7 - flat line of data points, does not show any curve

A8 - flat line of data points, does not show any curve

B8 - flat line of data points, does not show any curve

C1 - flat line of data points, does not show any curve

C2 - flat line of data points, does not show any curve

C8 - flat line of data points, does not show any curve

D2 - flat line of data points, does not show any curve

G1 - flat line of data points, does not show any curve

G2 - flat line of data points, does not show any curve

H1 - flat line of data points, does not show any curve

J8 - flat line of data points, does not show any curve

K1 - flat line of data points, does not show any curve

K5 - flat line of data points, does not show any curve

K6 - flat line of data points, does not show any curve

L1 - flat line of data points, does not show any curve, with disconnected data points

L2 - flat line of data points, does not show any curve

L5 - flat line of data points, does not show any curve

L6 - flat line of data points, does not show any curve

P1 - flat line of data points, does not show any curve

P5 - flat line of data points, does not show any curve

P6 - flat line of data points, does not show any curve

E3 - noisy data, multiple unexplained outliers

F3 - noisy data, multiple unexplained outliers

H2 - flat line of data points, does not show any curve

I2 - noisy data points, multiple unexplained outliers, near straight diagonal line

I3 - noisy data points, multiple unexplained outliers

J3 - noisy data points, multiple unexplained outliers

M1 - flat line of data points, does not show any curve

N3 - noisy data points, multiple unexplained outliers

BDH Bilal - Dataset series Z1

A6 - noisy data points, multiple unexplained outliers

A10 - noisy data points, straight diagonal line

A11 - noisy data points, straight diagonal line

A12 - noisy data points, straight diagonal line

B6 - noisy data points

D3 - noisy data points, unexplained flat line of data points

E7 - multiple curvatures from datapoints, cannot be explained with mechanistic rate law

E8 - multiple curvatures from datapoints, cannot be explained with mechanistic rate law

F11- noisy data points, cannot be explained with mechanistic rate law

F12 - multiple curvatures from datapoints, cannot be explained with mechanistic rate law

H1 - noisy data points, cannot be explained with mechanistic rate law

H7 - multiple curvatures from datapoints, cannot be explained with mechanistic rate law

H8 - multiple curvatures from datapoints, cannot be explained with mechanistic rate law

L10 - noisy data points, cannot be explained with mechanistic rate law

L9 - noisy data points, cannot be explained with mechanistic rate law

L12 - multiple curvatures from datapoints, cannot be explained with mechanistic rate law

A9 - flat line of data points

F8 - multiple curvatures from datapoints, cannot be explained with mechanistic rate law

F4 - multiple curvatures from datapoints, cannot be explained with mechanistic rate law

F10 - multiple curvatures from datapoints, cannot be explained with mechanistic rate law

F11 - multiple curvatures from datapoints, difficulty in fitting due to differences in initial condition and experimental measurement

F12 - multiple curvatures from datapoints, cannot be explained with mechanistic rate law

G8 - multiple curvatures from datapoints, difficulty in fitting due to differences in initial condition and experimental measurement

G9 - multiple curvatures from datapoints, difficulty in fitting due to differences in initial condition and experimental measurement

G10 - multiple curvatures from datapoints, difficulty in fitting due to differences in initial condition and experimental measurement

G11 - multiple curvatures from datapoints, difficulty in fitting due to differences in initial condition and experimental measurement

G12 - multiple curvatures from datapoints, difficulty in fitting due to differences in initial condition and experimental measurement

H1 - multiple curvatures from datapoints, cannot be explained with mechanistic rate law

H7 - multiple curvatures from datapoints, cannot be explained with mechanistic rate law

K12 - multiple curvatures from datapoints, cannot be explained with mechanistic rate law

K4 - multiple curvatures from datapoints, cannot be explained with mechanistic rate law
