# Supplementary material for: Parameterization of cell-free systems with time-series data using KETCHUP
Source: PLoS Comput Biol. 2025 Nov 21;21(11):e1013724. doi: 10.1371/journal.pcbi.1013724 (PMC12637948; doi:10.1371/journal.pcbi.1013724)
Supplement: S2 Text — (DOCX) [file pcbi.1013724.s014.docx]

The extension of KETCHUP was used to parameterize time course NADH data for FDH and BDH and is described below. To help in summarizing the dynamic parameterization process we define the following sets and variables:

**Sets**
Set of datasets K = {*k* ∀ *k* = 1, … , n_k_}for n_k_ datasets

**Parameters**t_k_ – discrete timepoints given for dataset *k*, excluding the initial point
t_0,k_ - denotes the initial time point for dataset *k*
$y_{k}^{meas}\left( t_{k} \right)$- measured NADH concentration for dataset k at time t
$k_{dQ}$ – NADH decomposition rate constant which is set during parameterization of FDH and BDH
E^FDH^ – enzyme FDH
E^BDH^ – enzyme BDH
R – Boltzmann’s gas constant (8.314 J/mol K)
T - temperature set at 310.15 K
$\Delta G^{\circ}$ - standard Gibbs free energy (-22.5 ± 4.1 kJ/mol)

**Variables**
$K_{I}^{A}$ – Inhibitor constant for NAD^+^$K_{I}^{Q}$ – Inhibitor constant for NADH
$K_{M}^{B}$ – Michaelis-Menten constant for formate
$K_{M}^{A}$ - Michaelis-Menten constant for NAD^+^$k_{cat}$ – turnover number (used for FDH)
$K_{M}^{P}$ – Michaelis-Menten constant for 2,3-butanediol
$K_{M}^{S}$ – Michaelis-Menten constant for acetoin
$K_{M}^{Q}$- Michaelis-Menten constant NADH
$k_{cat}^{f}$ – forward turn over number (used for BDH)
$k_{cat}^{r}$ – backward turn over number (used for BDH)
$y_{k}^{pred}\left( t_{k} \right)$ – predicted NADH concentration for dataset k at time t
A – metabolite NAD^+^B – metabolite formate
Q – metabolite NADH
S – metabolite acetoin
P – metabolite 2,3-butanediol

The objective function of the optimization problem aims to minimize the squared distance between measured and experimental reaction flux at each discrete time point (t) with each point weighed equally (*i.e.,* 1). The formulation for the sum of squared residuals (SSR) is described as:

Minimize: $SSR=\sum_{k\in K} \frac{\left( y_{k}^{meas}\left( t_{k} \right)-y_{k}^{pred}\left( t_{k} \right) \right)^{2}}{1}$

There are two different set of constraints used: *(a)* one for the parameterization of FDH and *(b)* the other for the parameterization of BDH.

(a) For FDH the formulation is constrained as such:

Subject to:

$$v(FDH)=\frac{k_{cat} \left[ A \right]\left[ B \right]}{K_{I}^{A}K_{M}^{B}+K_{M}^{B}\left[ A \right]+K_{M}^{A}\left[ B \right]+\left[ A \right]\left[ B \right]+\frac{K_{I}^{A}K_{M}^{B}}{K_{I}^{Q}}\left[ Q \right]+\frac{K_{M}^{A}}{K_{I}^{Q}}[B][Q]}[E^{FDH}]$$

$$v\left( NADH_{decomp} \right)=-k_{dQ}[Q]$$

$$\frac{d\left[ A \right]}{dt}=-v(FDH)$$

$$\frac{d\left[ B \right]}{dt}= -v(FDH)$$

$$\frac{d\left[ Q \right]}{dt}=v(FDH)- v\left( NADH_{decomp} \right)$$

$$A\left( 0 \right)=y_{k}^{meas}\left( t_{0,k} \right)$$

$$y_{k\left( t_{k} \right)}^{pred}=A(t)$$

$$A\left( t \right),B\left( t \right),Q\left( t \right)\geq0$$

(b) For BDH the formulation is constrained as such:

Subject to:

$$v\left( BDH \right)=\frac{\frac{k_{cat}^{f}\left[ S \right]\left[ Q \right]}{K_{M}^{S}K_{M}^{Q}}-\frac{k_{cat}^{r}\left[ P \right]\left[ A \right]}{K_{M}^{P}K_{M}^{A}}}{\left( 1+\frac{\left[ S \right]}{K_{M}^{S}} \right)\left( 1+\frac{\left[ Q \right]}{K_{M}^{Q}} \right)+\left( 1+\frac{\left[ P \right]}{K_{M}^{P}} \right)\left( 1+\frac{\left[ A \right]}{K_{M}^{A}} \right) -1}[E^{BDH}]$$

$$v\left( NADH_{decomp} \right)=-k_{dQ}[Q]$$

$$\frac{d\left[ Q \right]}{dt}=-v\left( BDH \right)- v\left( NADH_{decomp} \right)$$

$$\frac{d\left[ S \right]}{dt}= -v\left( BDH \right)$$

$$\frac{d\left[ A \right]}{dt}=v\left( BDH \right)$$

$$\frac{d\left[ P \right]}{dt}=v\left( BDH \right)$$

$$A\left( 0 \right)=y_{k}^{meas}\left( t_{0,k} \right)$$

$$y_{k\left( t_{k} \right)}^{pred}=A(t)$$

$$K_{eq}=\frac{K_{cat}^{f}K_{M}^{P}K_{M}^{A}}{K_{cat}^{r}K_{M}^{S}K_{M}^{Q}}=e^{-\frac{\Delta G^{\circ}}{RT}}$$

$$A\left( t \right),S\left( t \right),Q\left( t \right),P(t)\geq0$$

Model discrimination

To determine significance of the time-lag adjustment, we use an adjusted Bartlett’s χ^2^-test to determine if the models (with and without time-lag adjustment) parameterized are independent from each other. We used equation (5) from Heijnen 2003[1], a study focusing on model discrimination of kinetic models shown below, we use variance of the SSR of all solutions found for s^2^:

$$T=\frac{\sum_{m=1}^{M} \left( n-p_{m} \right)\ln\left( \frac{s_{tot}^{2}}{s_{m}^{2}} \right)}{1+\frac{1}{3\left( M-1 \right)}\left[ \sum_{m=1}^{M} \frac{1}{n-p_{m}}-\frac{1}{\sum_{m=1}^{M} \left( n-p_{m} \right)} \right]}\sim\chi2\left( M-1 \right)$$

Variable values are listed below:

|  |  |  |  | With time delay | | Without time delay | |  |
| --- | --- | --- | --- | --- | --- | --- | --- | --- |
| Model name | M  # Models | n  # datasets | $s_{tot}^{2}$ | P_m_  # parameters | $s_{m}^{2}$ | P_m_  # parameters | $s_{m}^{2}$ | T |
| FDH – B1 | 2 | 19 | 0.109 | 8 | 0.00569 | 7 | 0.0860 | 33.8 |
| FDH – B2 | 2 | 59 | 9.89 | 8 | 1.86 | 7 | 7.82 | 96.5 |
| BDH – Z1 | 2 | 78 | 8.06 | 9 | 0.068 | 8 | 10.3 | 310.1 |

χ^2^(df= 1, α=0.001) = 10.828

Test scores for all models are greater than Bartlett’s test score at α = 0.001, indicating significance between models.

1. Verheijen PJT. Model selection: An overview of practices in chemical engineering. 2003. pp. 85–104. doi:10.1016/S1570-7946(03)80071-8
