# Supplementary material for: Parameterization of cell-free systems with time-series data using KETCHUP
Source: PLoS Comput Biol. 2025 Nov 21;21(11):e1013724. doi: 10.1371/journal.pcbi.1013724 (PMC12637948; doi:10.1371/journal.pcbi.1013724)
Supplement: S4 File — Fig A. raw data fitting for Dataset B1. Fig B. raw data fitting for Dataset B2. Fig C. raw data fitting for Dataset Z1. Fig D. simulation of best solution for Dataset B1 with and without time-lag. Fig E. simulation of best solution for Dataset B2 with and without time-lag. Fig F. simulation of best solution for Dataset B1 with and without time-lag. (DOCX) [file pcbi.1013724.s018.docx]

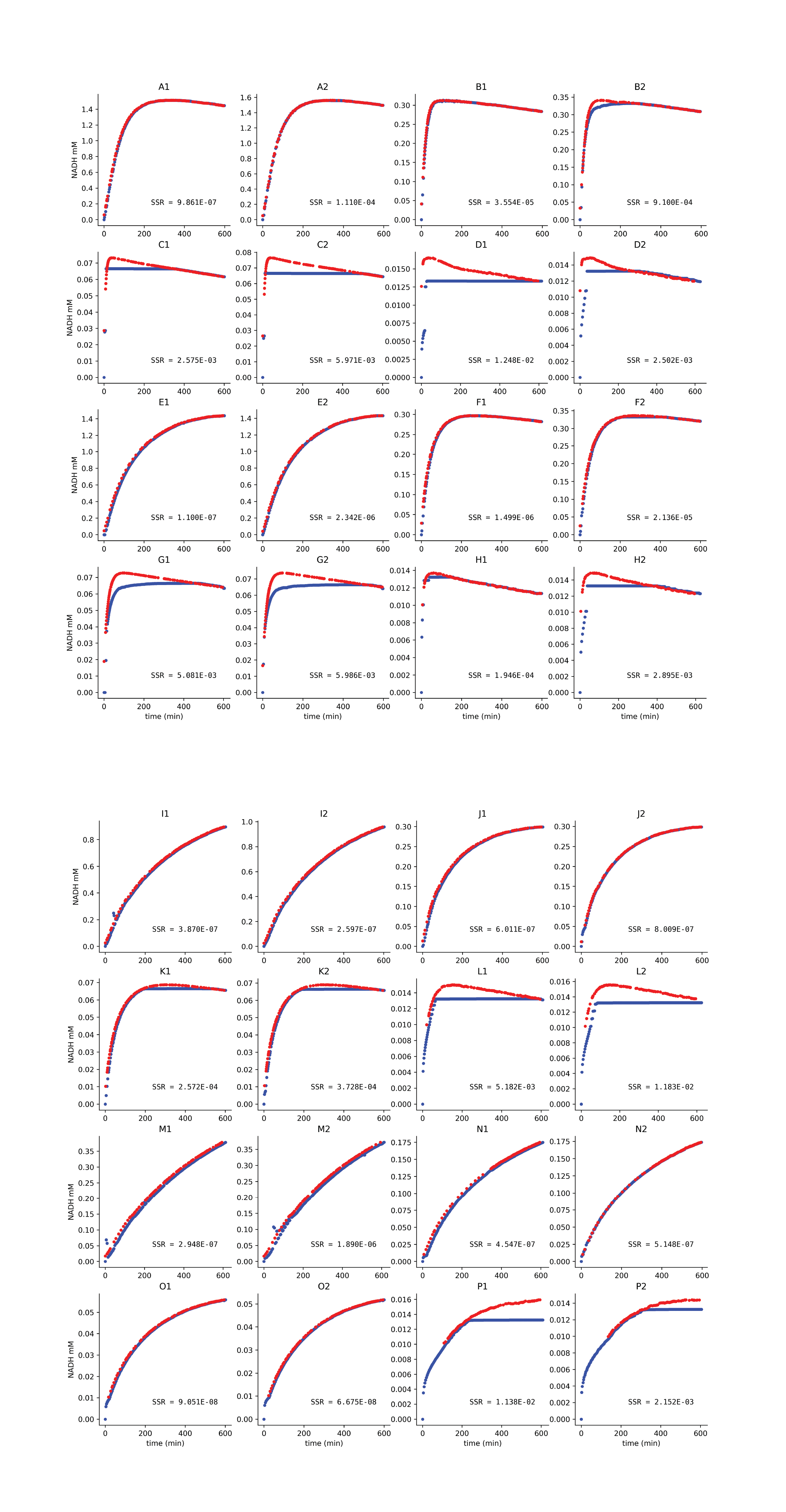


*Fig A: Raw data (red dots) and predicted (blue dots) for Formate dehydrogenase dataset B1. Each plot is labeled with their respective well id and SSR fit value from parameterization of the single dataset.*


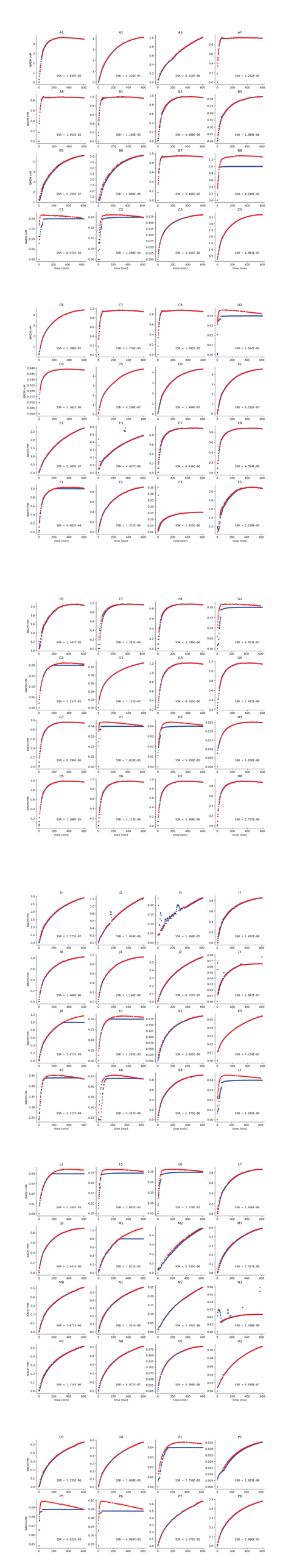


*Fig B: Raw data (red dots) and predicted (blue dots) for Formate dehydrogenase dataset B2. Each plot is labeled with their respective well id and SSR fit value from parameterization of the single dataset.*


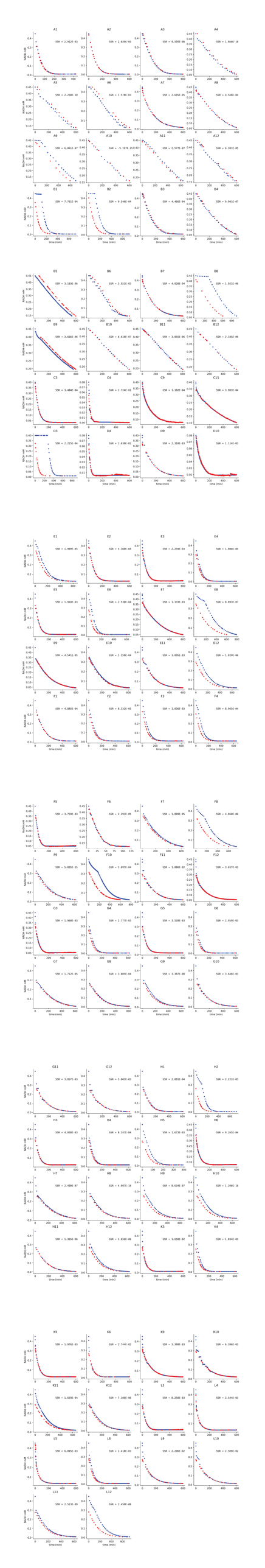


*Fig C: Raw data (red dots) and predicted (blue dots) for 2,3-butanediol dehydrogenase dataset Z1. Each plot is labeled with their respective well id and SSR fit value from parameterization of the single dataset.*


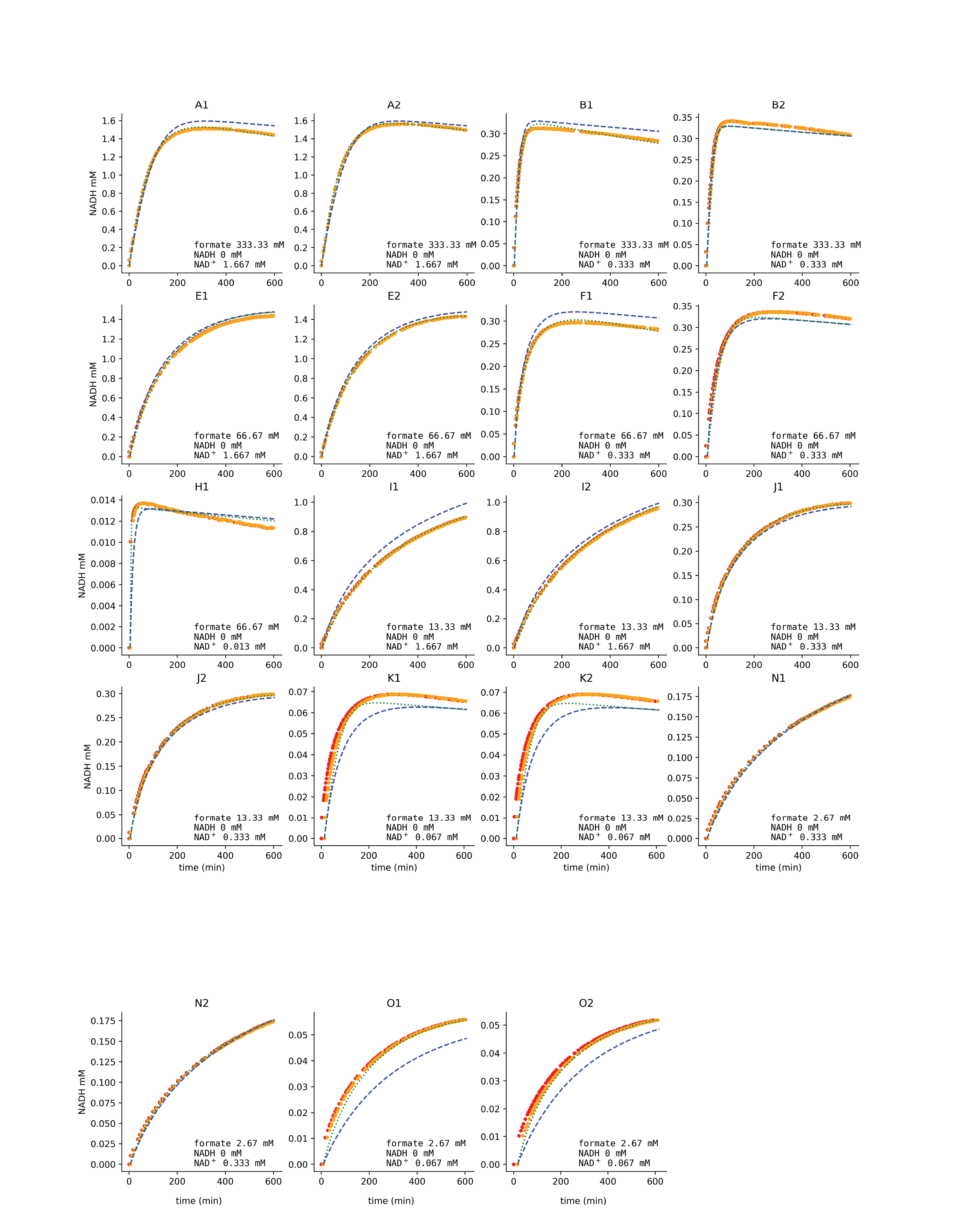
*Fig D: Simulation of NADH concentration for FDH dataset B1 over time between experimental (red dots), experimental with adjusted time delay (orange dots), simulation of separately parameterized dataset (dotted green line), and simulation of combined datasets (dashed blue line). Plot contains text of initial metabolite information and respective experimental well as title.*


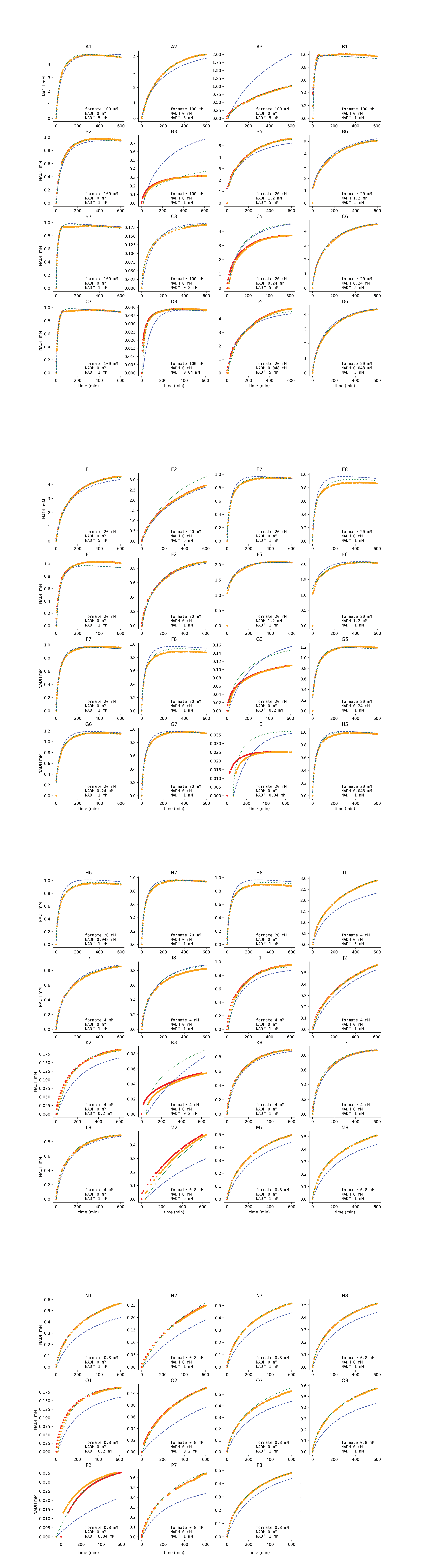
*Fig E: Simulation of NADH concentration for FDH dataset B2 over time between experimental (red dots), experimental with adjusted time delay (orange dots), simulation of separately parameterized dataset (dotted green line), and simulation of combined datasets (dashed blue line). Plot contains text of initial metabolite information and respective experimental well as title.*


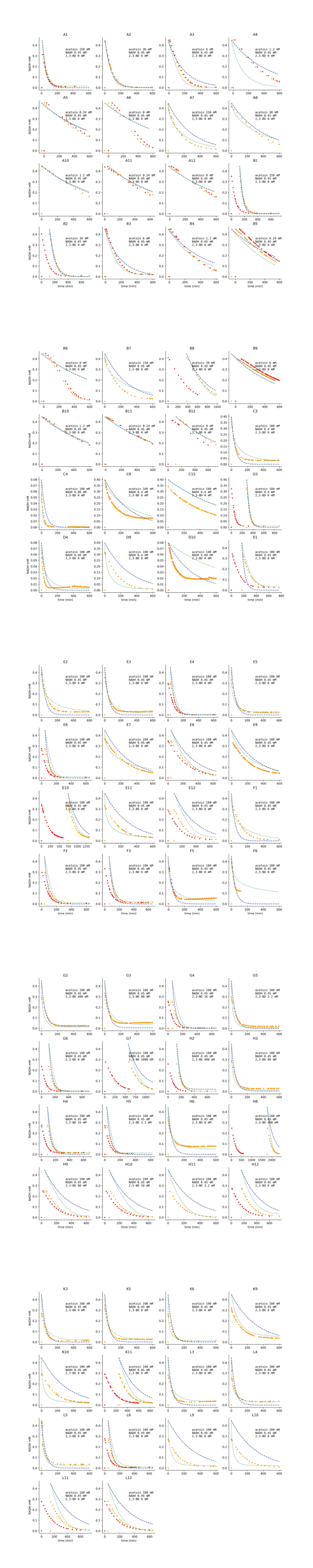
*Fig F: Simulation of NADH concentration for BDH dataset Z1 over time between experimental (red dots), experimental with adjusted time delay (orange dots), simulation of separately parameterized dataset (dotted green line), and simulation of combined datasets (dashed blue line). Plot contains text of initial metabolite information and respective experimental well as title.*
